# Chemically Aware Model Builder (camb): an R package for property and bioactivity modelling of small molecules

**DOI:** 10.1186/s13321-015-0086-2

**Published:** 2015-08-28

**Authors:** Daniel S Murrell, Isidro Cortes-Ciriano, Gerard J P van Westen, Ian P Stott, Andreas Bender, Thérèse E Malliavin, Robert C Glen

**Affiliations:** Department of Chemistry, Centre for Molecular Informatics, University of Cambridge, Lensfield Road, Cambridge, CB2 1EW UK; Unite de Bioinformatique Structurale, Structural Biology and Chemistry Department, Institut Pasteur and CNRS UMR 3825, 25-28, rue Dr. Roux, 75 724 Paris, France; European Molecular Biology Laboratory, European Bioinformatics Institute, Wellcome Trust Genome Campus, Hinxton, CB101SD UK; Unilever Research, Port Sunlight Laboratory, Bebington, L63 3JW Wirral UK

**Keywords:** R, Package, Ensemble, Learning, Workflow, QSPR, QSAR, PCM

## Abstract

**Background:**

In silico predictive models have proved to be valuable for the optimisation of compound potency, selectivity and safety profiles in the drug discovery process.

**Results:**

*camb* is an R package that provides an environment for the rapid generation of quantitative Structure-Property and Structure-Activity models for small molecules (including QSAR, QSPR, QSAM, PCM) and is aimed at both advanced and beginner R users. *camb's* capabilities include the standardisation of chemical structure representation, computation of 905 one-dimensional and 14 fingerprint type descriptors for small molecules, 8 types of amino acid descriptors, 13 whole protein sequence descriptors, filtering methods for feature selection, generation of predictive models (using an interface to the R package *caret*), as well as techniques to create model ensembles using techniques from the R package *caretEnsemble*). Results can be visualised through high-quality, customisable plots (R package *ggplot2*).

**Conclusions:**

Overall, *camb* constitutes an open-source framework to perform the following steps: (1) compound standardisation, (2) molecular and protein descriptor calculation, (3) descriptor pre-processing and model training, visualisation and validation, and (4) bioactivity/property prediction for new molecules. *camb* aims to speed model generation, in order to provide reproducibility and tests of robustness. QSPR and proteochemometric case studies are included which demonstrate *camb's* application.

**Graphical abstract:**
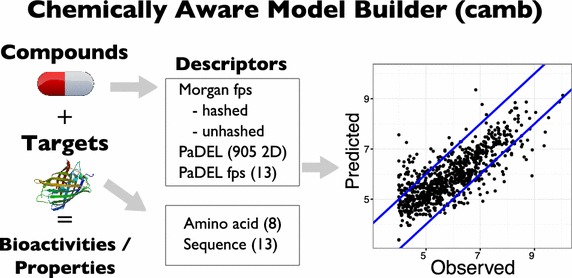
From compounds and data to models: a complete model building workflow in one package.

**Electronic supplementary material:**

The online version of this article (doi:10.1186/s13321-015-0086-2) contains supplementary material, which is available to authorized users.

## Background

The advent of high-throughput technologies over the last two decades has led to a vast increase in the number of compound and bioactivity databases [1–3]. This increase in the amount of chemical and biological information has been exploited by developing fields in drug discovery such as quantitative structure activity relationships (QSAR), quantitative structure property relationships (QSPR), quantitative sequence-activity modelling (QSAM), or proteochemometric modelling (PCM) [4, 5].

**Fig. 1:**
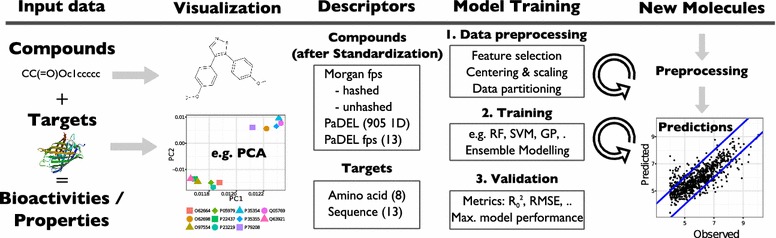
Overview of camb functionalities. *camb* provides an open and seamless framework for bioactivity/property modelling (QSAR, QSPR, QSAM and PCM) including: (1) compound standardisation, (2) molecular and protein descriptor calculation, (3) pre-processing and feature selection, model training, visualisation and validation, and (4) bioactivity/property prediction for new molecules. In the first instance, compound structures are subjected to a common representation with the function *StandardiseMolecules*. Proteins are encoded with 8 types of amino acid and/or 13 types of full protein sequence descriptors, whereas *camb* enables the calculation of 905 1D physicochemical descriptors for small molecules, and 14 types of fingerprints, such as Morgan or Klekota fingerprints. Molecular descriptors are statistically pre-processed, e.g., by centering their values to zero mean and scaling them to unit variance. Subsequently, single or ensemble machine learning models can be trained, visualised and validated. Finally, the *camb* function *PredictExternal* allows the user (1) to read an external set of molecules with a trained model, (2) to apply the same processing to these new molecules, and (3) to output predictions for this external set. This ensures that the same standardization options and descriptor types are used when a model is applied to make predictions for new molecules.

The R programming environment provides a flexible and open platform for statistical analyses [6]. R is extensively used in genomics [7], and the availability of R packages for cheminformatics and medicinal chemistry is small in comparison. Nonetheless, R currently constitutes the most frequent choice in the medicinal chemistry literature for compound bioactivity and property modelling [8]. In general, these studies share a common algorithmic structure, which can be summarised in four model generation steps: (1) compound standardisation, (2) descriptor calculation, (3) pre-processing, feature selection, model training and validation, and (4) bioactivity/property prediction for new molecules. Fig. 1 illustrates these steps.

Currently available R packages provide the capability for only subsets of the above mentioned steps. For instance, the R packages *chemmineR* [9] and *rcdk* [10] enable the manipulation of SDF and SMILES files, the calculation of physicochemical descriptors, the clustering of molecules, and the retrieval of compounds from PubChem [3]. On the machine learning side, the *caret* package provides a unified platform for the training of machine learning models [11].

While it is possible to use a combination of these packages to set up a desired workflow, going from start to finish requires a reasonable understanding of model building in *caret*.

Here, we present the R package *camb*: *C*hemically *A*ware *M*odel *B*uilder, which aims to address the current lack of an R framework comprising the four steps mentioned above. Specifically, the *camb* package makes it extremely easy to enter new molecules (that have no previous standardisation) through a single function, to acquire new predictions once model building has been done. The package has been conceived such that users with minimal programming skills can generate competitive predictive models and high-quality plots showing the performance of the models under default operation. It must be noted that *camb* does limit practitioners to a limited but easily used workflow to begin with. Experienced users, or those that intend to practice machine learning in R extensively are encouraged to neglect this basic wrapper completely on their second training attempt and learn how to use the *caret* package from the *caret* related vignettes directly.

Overall, *camb* enables the generation of predictive models, such as Quantitative Structure–Activity Relationships (QSAR), Quantitative Structure–Property Relationships (QSPR), Quantitative Sequence–Activity Modelling (QSAM), or Proteochemometric Modelling (PCM), starting with: chemical structure files, protein sequences (if required), and the associated properties or bioactivities. Moreover, *camb* is the first R package that enables the manipulation of chemical structures utilising Indigo’s C API [12], and the calculation of: (1) molecular fingerprints and 1-D [13] topological descriptors calculated using the PaDEL-Descriptor Java library [14], (2) hashed and unhashed Morgan fingerprints [15], and (3) eight types of amino acid descriptors. Two case studies illustrating the application of *camb* for QSPR modelling (solubility prediction) and PCM are available in the Additional files 1, 2.

## Design and implementation

This section describes the tools provided by *camb* for (1) compound standardisation, (2) descriptor calculation, (3) pre-processing and feature selection, model training, visualisation and validation, and (4) bioactivity/property prediction for new molecules.

### Compound standardization

Chemical structure representations are highly ambiguous if SMILES are used for representation—for example, when one considers aromaticity of ring systems, protonation states, and tautomers present in a particular environment. Hence, standardisation is a step of crucial importance when either storing structures or before descriptor calculation. Many molecular properties are dependent on a consistent assignment of the above criteria in the first place. If one examines large chemical databases one can see how important this step is—a rather good explanation for standardisation is found in PubChem, one of the largest public databases, can be found on the PubChem Blog [16]. Hence, we are of the opinion that standardising chemical structures is crucial in order to provide consistent data for later modelling steps, in line with perceptions by others (such as the PubChem curators). For standardisation, *camb* provides the function *StandardiseMolecules* which utilises Indigo’s C API [12]. SDF and SMILES formats are provided as molecule input options. Any molecules that Indigo fails to parse are removed during the standardisation step. As a filter, the user can stipulate the maximum number of each halogen atom that a compound can possess in order to pass the standardisation process. This allows datasets with a bias towards many molecules that contain one type of halogen to be easily normalised before training. Additional arguments of this function include the removal of inorganic molecules or those compounds with a molecular mass above or below a defined threshold. Most importantly, *camb* makes use of Indigo’s InChI [17] plugin to represent all tautomers by the same canonical SMILES by converting molecules to InChI, discarding tautomeric information, and converting back to SMILES.

### Descriptor calculation

Currently, *camb* supports the calculation of compound descriptors and fingerprints via PaDEL-Descriptor [14], and Morgan circular fingerprints [15] as implemented in RDkit [18]. The function *GeneratePadelDescriptors* permits the calculation of 905 1- and 2-D descriptors and 10 PaDEL-Descriptor fingerprints, namely: CDK fingerprints [19], CDK extended fingerprints [19], Kier-Hall E-state fragments [20], CDK graph only fingerprints [19], MACCS fingerprints [21], Pubchem fingerprints [3], Substructure fingerprints [22], and Klekota–Roth fingerprints [23].

In addition to the PaDEL-Descriptor fingerprints, Morgan fingerprints can be computed with the function *MorganFPs* through the python library RDkit [18]. Hashed fingerprints can be generated as *binary*, recording the presence or absence of each substructure, or *count based*, recording the number of occurrences of each substructure. Additionally, the *MorganFPs* function also computes unhashed (keyed) fingerprints, where each substructure in the dataset is assigned a unique position in a binary fingerprint of length equal to the number of substructures existing in the dataset. Since the positions of substructures in the unhashed fingerprint depend on the dataset, the function *MorganFPs* allows calculation of unhashed fingerprints for new compounds using a basis defined by the substructures present in the training dataset. This ensures that substructures in new compounds map to the same locations on the fingerprint and allows enhanced model interpretation by noting which exact substructures are deemed important by the learning algorithm.

The function *SeqDescs* enables the calculation of 13 types of whole protein sequence descriptors from UniProt identifiers or from amino acid sequences [24], namely: amino acid composition (AAC), dipeptide composition (DC), tripeptide composition (TC), normalized Moreau–Broto autocorrelation (MoreauBroto), Moran autocorrelation (Moran), Geary autocorrelation (Geary), CTD (composition/transition/distribution) (CTD), Conjoint Traid (CTriad), sequence order coupling number (SOCN), quasi-sequence order descriptors (QSO), pseudo amino acid composition (PACC), amphiphilic pseudo amino acid composition (APAAC) [25, 26].

In addition, *camb* permits the calculation of 8 types of amino acid descriptors, namely: 3 and 5 Z-scales (Z3 and Z5), T-Scales (TScales), ST-Scales (STScales), Principal Components Score Vectors of Hydrophobic, Steric, and Electronic properties (VHSE), BLOSUM62 Substitution Matrix (BLOSUM), FASGAI (FASGAI), MSWHIM (MSWHIM), and ProtFP PCA8 (ProtFP8). Amino acid descriptors can be used for modelling of the activity of small peptides or for the description of protein binding sites [5, 25, 27, 28]. Multiple sequence alignment gaps are supported by this *camb* functionality. Descriptor values for these gaps are encoded with zeros. Further details about these descriptors and their predictive signal for bioactivity modelling can be found in two recent publications [25, 26].

### Model training and validation

Prior to model training, descriptors often need to be pre-processed [29] so that they are equally weighted as inputs into the learning algorithms and to remove any that contain little relevant information content. To this end, several functions (see package documentation and tutorials) are provided. These functions include the removal of non-informative descriptors (function *RemoveNearZeroVarianceFeatures*) or highly correlated descriptors (function *RemoveHighlyCorrelatedFeatures*), the imputation of missing descriptor values (function *ImputeFeatures*), and descriptor centering and scaling to unit variance (function *PreProcess*) among others [30].

The R package *caret* provides a common interface to the most popular machine learning packages that exist in R, and, as such, *camb* invokes *caret* to set up cross-validation frameworks and train machine learning models. These include learning methods in Bagging, Bayesian Methods, Boosting, Boosted Trees, Elastic Net, MARS, Gaussian Processes, K Nearest Neighbour, Principal Component Regression, Radial Basis Function Networks, Random Forests, Relevance Vector Machines, and Support Vector Machines among others. Additionally, two ensemble modelling approaches, namely greedy and stacking optimisation, have been integrated from the R package *caretEnsemble* [31], which allows the combination of models to form ensemble models, which have proven to be less error prone [28].

In greedy optimization [32], the cross-validated RMSE is optimized using a linear combination of input model predictions. The input models are all trained using an identical fold composition. Each model is assigned a weight in the following manner. Initially, all models have their weight set to zero. The weight for a given model is repeatedly incremented by 1 if the subsequent normalized weight vector results in a closer match between the weighted combination of cross-validated predictions and the observed values (i.e. lower RMSE of the linear combination). This repetition is carried out *n* times, by default *n* = 1,000. The resulting weight vector is then normalized to obtain a final weight vector.

In the case of model stacking [28], the predictions of the input models serve as training data points for a meta-model. This meta-model can have linear, e.g. Partial Least Squares [33], or non-linear, *e.g.* Random Forest [34] characteristics. If the selected algorithm allows the importance of its inputs to be determined, each input corresponds to a single model, then the relative contributions of each model to the prediction can be ascertained. These model ensembles can be applied to a test set (which was not used when building the ensembles), and the error metric (e.g. RMSE) compared to that of the single models on the test set.

In the general case, prior to model training, the dataset is divided into a training set, comprising e.g. 70% of the data, and a test set, which comprises the remaining data. The test set is used to assess the predictive power of the models on new data points not considered in the training phase. In the training phase, the values of the model parameters (hyper-parameters) are optimized by grid search and *k*-fold cross-validation (CV) [35]. A grid of plausible hyper-parameter values covering an exponential range is defined (function *expGrid*). Next, the training set is split into *k* folds by, e.g. stratified or random sampling of the bioactivity/property values. For each combination of hyper-parameters, a model is trained on $k-1$ folds, and the values for the remaining fold are then predicted. This procedure is repeated *k* times, each time holding out a different fold. The values of the hyper-parameters exhibiting the lowest average RMSE (or another metric such as e.g. *R*^2^) value across the *k* folds are considered optimal. A model is then trained on the whole training set using the optimal hyper-parameter values, and the predictive power of this model is assessed on the test set. The final model, trained on the whole dataset after having optimized the hyper-parameter values by CV, can be used to make predictions on an external chemical library.

Statistical metrics for model validation have also been included:

*During cross-validation*

1 $$q_{{CV}}^{2} \;or\;R_{{CV}}^{2} = 1 - \frac{{\sum\nolimits_{{i = 1}}^{{N_{{tr}} }} {(y_{i} - {\text{ }}\tilde{y}_{i} )^{2} } }}{{\sum\nolimits_{{i = 1}}^{{N_{{tr}} }} {(y_{i} - \bar{y_{tr}} )^{2} } }}{\text{ }}$$

2 $$\begin{aligned} RMSE_{CV} = \sqrt{\frac{(y_i - \widetilde{y}_i)^{2}}{N}} \end{aligned}$$

where $N_{tr}$, $y_i$, $\widetilde{y}_i$ and $\bar{y_{tr}}$ represent the size of the training set, observation *i*, prediction *i*, and the average value of observations in the training set, respectively.

*During testing*

3 $$Q_{{1\;test}}^{2} = 1 - \frac{{\sum\nolimits_{{j = 1}}^{{N_{{test}} }} {(y_{j} - \tilde{y}_{j} )^{2} } }}{{\sum\nolimits_{{j = 1}}^{{N_{{test}} }} {(y_{j} - \bar{y_{tr}} )^{2} } }}$$

4 $$Q_{{2\;test}}^{2} = 1 - \frac{{\sum\nolimits_{{j = 1}}^{{N_{{test}} }} {(y_{j} - \tilde{y}_{j} )^{2} } }}{{\sum\nolimits_{{j = 1}}^{{N_{{test}} }} {(y_{j} - \bar{y_{test}} )^{2} } }}$$

5 $$Q_{{3\;test}}^{2} = 1 - \frac{{{{\left[ {\sum\nolimits_{{j = 1}}^{{N_{{test}} }} {(y_{j} - \tilde{y}_{j} )^{2} } } \right]} \mathord{\left/ {\vphantom {{\left[ {\sum\nolimits_{{j = 1}}^{{N_{{test}} }} {(y_{j} - \tilde{y}_{j} )^{2} } } \right]} {N_{{test}} }}} \right.} {N_{{test}} }}}}{{{{\left[ {\sum\nolimits_{{j = 1}}^{{N_{{tr}} }} {(y_{j} - \bar{y_{tr}} )^{2} } } \right]} \mathord{\left/ {\vphantom {{\left[ {\sum\nolimits_{{j = 1}}^{{N_{{tr}} }} {(y_{j} - \bar{y_{tr}} )^{2} } } \right]} {N_{{tr}} }}} \right.} {N_{{tr}}}}}}$$

6 $$\begin{aligned} RMSE_{test} = \sqrt{\frac{(y_j - \widetilde{y}_j)^{2}}{N}} \end{aligned}$$

7 $$R_{{test}} = \frac{{\sum\nolimits_{{j = 1}}^{{N_{{test}} }} {\left(y_{j} - \bar{y_{test}} \right)} \left(\tilde{y}_{j} - \mathop {\bar{\tilde{y}_{{test}} }}\nolimits \right)}}{{\sqrt {\sum\nolimits_{{j = 1}}^{{N_{{test}} }} {\left(y_{j} - \bar{y_{test}} \right)^{2} } \sum {\left(\tilde{y}_{j} - \mathop {\bar{\tilde{y}_{{test}} }}\nolimits \right)^{2} } } }}$$

8 $$R_{{0\;test}}^{2} = 1 - \frac{{\sum\nolimits_{{j = 1}}^{{N_{{test}} }} {\left(y_{j} - {\text{ }}\tilde{y}_{j}^{{r0}} \right)^{2} } }}{{\sum\nolimits_{{j = 1}}^{{N_{{test}} }} {\left(y_{j} - \bar{y_{test}} \right)^{2} } }}$$

where $N_{tr}$, $N_{test}$, $y_j$, $\widetilde{y}_j$, and $\bar{y_{test}}$ represent the size of the training and test sets, observation *j*, prediction *j*, and the average value of observations in the test set, respectively. $\bar{y_{tr}}$ represents the average value of observations in the training set.

$R_{0\ test}^2$ is the square of the coefficient of determination through the origin, being $\widetilde{y}_{j}^{ r0} = k \widetilde{y}_j$ the regression through the origin (observed versus predicted) and *k* its slope. The reader is referred to Ref. [36] for a detailed discussion of both the evaluation of model predictive ability through the test set and about the three different formulations for $Q^{2}_{test}$, namely $Q_{1\ { test}}^{2}$, $Q_{2\ { test}}^{2}$, and $Q_{3\ { test}}^{2}$. The value of these metrics permits the assessment of model performance according to the criteria proposed by Tropsha and Golbraikh [37, 38], namely: $q_{{ CV}}^{2} > 0.5$, $R_{test}^2 > 0.6$, $\frac{(R_{test}^2 - R_{0\ test}^2)}{R_{test}^2} < 0.1$, and $0.85 \le k \le 1.15$.

These values might change depending on the dataset modelled, as well as on the application context, e.g. higher errors might be tolerated in hit identification in comparison to lead optimization, nevertheless, these criteria can serve as general guidelines to assess model predictive ability. The function *Validation* permits the calculation of all these metrics.

In cases where information about the experimental error of the data is available, the values for the statistical metrics on the test set can be compared to the theoretical maximum and minimum achievable performance given (1) the uncertainty of the experimental measurements, (2) the size of the training and test sets, and (3) the distribution of the dependent variable [39]. The distribution of maximum and minimum $R_{0\ test}^2, R_{test}, Q^{2}_{test}$, and RMSE_test_ values can be computed with the functions *MaxPerf* and *MinPerf*. The distributions of maximum model performance are calculated in the following way. A sample, *S*, of size equal to the test set is randomly drawn from the dependent variable, e.g. IC_50_ values. Next, the experimental uncertainty is added to *S*, which defines the sample $S_{noise}$. The $R_{0\ test}^2, R_{test}, Q^{2}_{test}$, and RMSE_test_ values for *S* against $S_{noise}$ are then calculated. These steps are repeated *n* times, by default 1,000, to calculate the distributions of $R_{0\ test}^2, R_{test}, Q^{2}_{test}$, and RMSE_test_ values. To calculate the distributions of minimum model performance, the same steps are followed, with the exception that *S* is randomly permuted before calculating the values for the statistical metrics.

### Visualization

**Fig. 2:**
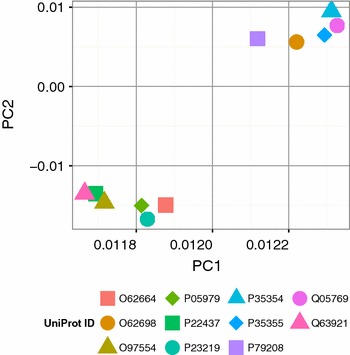
PCA analysis output from PCM. PCA analysis of the binding site amino acid descriptors corresponding to the 11 mammalian cyclooxygenases considered in the second case study (Proteochemometrics). Binding site amino acid descriptors (5 Z-scales) were input to the function *PCA*. The first two principal components (PCs) explained more than 80% of the variance. This indicates that there are mainly two sources of variability in the data. To generate the plot, we used the function *PCAPlot* using the default options. Cyclooxygenases cluster into two distant groups, which correspond to the isoenzyme type, i.e. *COX-1 and COX-2*. Given that small molecules tend to display similar binding profiles within orthologues [43], we hypothesised that merging bioactivity data from paralogues and orthologues will lead to more predictive PCM models [28].

Visualization functionality for model performance and for exploratory analyses of the data is provided. All plots are generated using the R package *ggplot2* [40]. Default options of the plotting functions were chosen to allow the generation of high-quality plots, and in addition, the layer-based structure of ggplot objects allows for further optimisation by the addition of customisation layers. The visualization tools include correlation plots (*CorrelationPlot*), bar plots with error bars (*ErrorBarplot*), and Principal Component Analysis (PCA) (*PCA* and *PCAPlot*), histograms (*DensityResponse*), and pairwise distance distribution plots (*PairwiseDistPlot*). For instance, the *camb* function *PCA* performs a Principal Component Analysis (PCA) on compound and/or protein descriptors. The output can be directly sent to the function *PCAPlot*, which will depict the two fist principal components, with the shape and color of a user-defined class *e.g.* compound class or protein isoform (Fig. 2).

Visual depiction of compounds is also possible with the function *PlotMolecules*, utilising Indigo’s C API. Visualization functions are exemplified in the tutorials provided in the Additional file 2 and with the package documentation (folder *camb/doc* of the package).

### Predictions for new molecules

One of the major benefits of having all tools available in one framework is that it is straightforward to perform exactly the same processing on new molecules as the ones used on the training set, e.g. standardisation of molecules and centering and scaling of descriptors. The *camb* function *PredictExternal* allows the user to read an external set of molecules together with a trained model, and outputs predictions on this external set. This *camb* functionality ensures that the same standardization options and descriptor types are used when a model is applied to make predictions for new molecules. An example of this is shown in the QSPR tutorial.

## Results and discussion

Two tutorials demonstrating property and bioactivity modelling are available as Additional files 1 and 2, and also within the package documentation. We encourage *camb* users to visit the package repository (https://github.com/cambDI/camb) for future updated versions of the tutorials. In the following subsections, we show the results obtained for the two case studies presented in the tutorials, namely: (1) QSPR: prediction of compound aqueous solubility (logS), and (2) PCM: modelling of the inhibition of 11 mammalian cyclooxygenases (COX) by small molecules. The datasets are available in the *examples/PCM* directory of the package. Further details about the PCM dataset can be found in Ref. [28].

### Case study 1: QSPR

To illustrate the functionalities of *camb* for compound property modelling, the aqueous solubility values for 1,708 small molecules were downloaded [41]. Aqueous solubility values were expressed as logS, where S corresponds to the solubility at a temperature of 20–25$^{\circ }$C in mol/L. A common representation for the compound structures was found using the function *StandardiseMolecules* with default parameters, meaning that all molecules were kept irrespective of their molecular mass or the number of halogens present within their structure. Molecules were represented with implicit hydrogens, dearomatized, and passed through the InChI format to ensure that tautomers were represented by the same SMILES. 905 one and two-dimensional topological and physicochemical descriptors were then calculated using the function *GeneratePadelDescriptors* provided by the PaDEL-Descriptor [14] Java library built into the *camb* package. Missing descriptor values were imputed with the function *ImputeFeatures*. Two filtering steps were then performed: (1) highly-correlated descriptors with redundant predictive signal were removed using the function *RemoveHighlyCorrelatedFeatures* with a cut-off value of 0.95, and (2) descriptors with near zero variance and hence limited predictive signal, were removed using the function *RemoveNearZeroVarianceFeatures* with a cut-off value of 30/1. Prior to model training, all descriptors were centered to have zero mean and scaled to have unit variance using the function *PreProcess*. After applying these steps the dataset consisted of 1,606 molecules encoded with 211 descriptors.

**Fig. 3:**
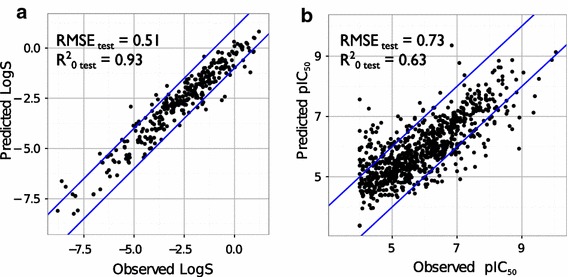
Observed vs predicted for both case studies. Observed against predicted values on the test set corresponding to **a** the compound solubility (LogS) dataset (case study 1: QSPR), and **b** the cyclooxygenase (COX) inhibition dataset (case study 2: PCM). Both **a** and **b** were generated with the function *CorrelationPlot*. The area defined by the* blue lines* comprises 1 LogS units (**a**) and 1 pIC_50_ units (**b**). Both* plots* were generated using the predictions on the test set calculated with the Linear Stacking ensembles (Tables 1, 3). Overall, high predictive power is attained on the test set for both datasets, with respective RMSE/$R_{0}^2$ values of 0.51/0.93 (**a**), and 0.73/0.63 (**b**). Taken together, these data indicate that ensemble modelling leads to higher predictive power, although this increase might be marginal for some datasets (**b**).

Three machine learning models were trained using 80% of the data (training set), namely: (1) Support Vector Machine (SVM) with a radial kernel, (2) Random Forest (RF), and (3) Gradient Boosting Machines (GBM). Fivefold cross-validation was used to optimize the value of the hyperparameters. Cross-validation and testing metrics for these three models are summarized in Table 1. Overall, the three algorithms displayed high performance on the test set, with RMSE/$R^{2}_{0}$ values of: GBM: 0.52/0.93; RF: 0.59/0.91; and SVM: 0.60/0.91 (Table 1; Fig. 3a). The combination of these three models as an ensemble was evaluated for improved predictive ability. To this end, two ensemble modelling techniques supported by *camb* were explored, namely: greedy optimization and model stacking. First, greedy ensemble was trained using the function *caretEnsemble* with 1,000 iterations. The greedy ensemble picked a linear combination of model outputs that was a local minimum in the RMSE landscape. Secondly, linear and non-linear stacking ensembles were created. In model stacking, the cross-validated predictions of a library of models are used as descriptors, on which a meta-model (ensemble model) is trained. This meta model can be a linear model, e.g. SVM with a linear kernel, or non linear, such as Random Forest. The application of ensemble modelling led to a decrease by 10–15% of RMSE_test_ values (Table 1). The highest predictive power was obtained with the greedy and the linear stacking ensembles, with $R^{2}_{0\ test}$/RMSE_test_ of 0.93/0.51 and 0.93/0.51, respectively. Taken together, these results indicate that higher predictive power can be obtained when modelling this dataset by combining different single QSPR models with either greedy optimisation or model stacking. From this case study it can be seen that by utilizing the *camb* package, a model training task which might involve porting datasets between multiple different external tools can be simplified to a few lines of code in a reproducible fashion within the R language alone. Additionally, predictions can easily be made on new molecules using a single function call passing in a new structures file.

**Table 1:** Cross-validation and testing metrics for the single and ensemble QSPR models trained on the compound solubility dataset

Algorithm		$R^{2}_{CV}$	RMSE_CV_	$R^{2}_{0\ test}$	RMSE_test_
A					
GBM		0.90	0.59	0.93	0.52
RF		0.89	0.62	0.91	0.59
SVM radial		0.88	0.63	0.91	0.60
B					
Greedy		–	0.57	0.93	0.51
Linear stacking		0.90	0.57	0.93	0.51
RF stacking		0.89	0.62	0.92	0.55

The lowest RMSE value on the test set, namely 0.51, was obtained with the greedy and with the linear stacking ensembles.

*GBM* Gradient Boosting Machine, *RF* Random Forest,* RMSE* root mean square error,* SVM* Support Vector Machine.

### Case study 2: proteochemometrics

In the second case study the functionalities of *camb* are illustrated for proteochemoemtric modelling. The tutorial “PCM with *camb*” (Additional file 2) reports the complete modelling pipeline for this dataset [28]. Bioactivity data for 11 mammalian COX (COX-1 and COX-2 inhibitors) was extracted from ChEMBL 16 [2, 28] (Table 2). Only the data satisfying the following criteria was kept: (1) assay score confidence higher than 8, (2) activity relationship equal to ‘=’, (3) activity type equal to “IC50”, and (4) activity unit equal to ‘nM’. The mean IC_50_ value was taken for duplicated compound-COX combinations. The final dataset comprised 3,228 distinct compounds and 11 mammalian COX proteins, with a total number of 4,937 datapoints (13.9% matrix completeness) [28].

**Table 2:** Cyclooxygenase inhibition dataset ("Results and discussion" section, case study 2)

UniProt ID	Isoenzyme	Organism	Number of datapoints
P23219	1	*Homo sapiens*	1,346
O62664	1	*Box taurus*	48
P22437	1	*Mus musculus*	50
O97554	1	*Oryctolagus cuniculus*	11
P05979	1	*Ovis aries*	442
Q63921	1	*Rattus norvegicus*	23
P35354	2	*Homo sapiens*	2,311
O62698	2	*Bos taurus*	21
Q05769	2	*Mus musculus*	305
P79208	2	*Ovis aries*	341
P35355	2	*Rattus norvegicus*	39

We extracted the bioactivity data for 11 mammalian cyclooxygenases from ChEMBL 16 [2]. The final bioactivity selection comprised 3,228 distinct compounds.

A common representation for the compound structures was found using the function *StandardiseMolecules* with default parameters. Then, two main descriptor types were calculated: (1) PaDEL descriptors [14] with the function *GeneratePadelDescriptors*, (2) and Morgan fingerprints with the function *MorganFPs*. Substructures with a maximal diameter of 4 bonds were considered. The length of the fingerprints was set to 512. To describe the target space, the binding site amino acid descriptors were derived from the crystallographic structure of ovine COX-1 complexed with celecoxib (PDB ID: 3KK6 [42]) by selecting those residues within a sphere of radius equal to 10 Å centered in the ligand. Subsequently, we performed multiple sequence alignment to determine the corresponding residues for the other 10 COX, and calculated 5 *Z*-scales for these residues with the function *AADescs*.

Prior to model training, missing descriptor values were imputed (function *ImputeFeatures*). Two filtering steps were then performed: (1) highly-correlated descriptors with redundant predictive signal were removed using the function *RemoveHighlyCorrelatedFeatures* with a cut-off value of 0.95, and (2) descriptors with near zero variance and hence limited predictive signal, were removed using the function *RemoveNearZeroVarianceFeatures* with a cut-off value of 30/1. Prior to model training, all descriptors were centered to have zero mean and scaled to have unit variance using the function *PreProcess*. These steps led to a final selection of 356 descriptors: 242 Morgan fingerprint binary descriptors, 99 physicochemical descriptors, and 15 *Z*-scales. The dataset was split into a training set, which was comprised of 80% of the data, and a test set (20%) with the function *SplitSet*. Three single PCM models were trained using fivefold cross-validation, namely: GBM, RF, and SVM with a radial kernel (Table 3).

**Table 3:** Cross-validation and testing metrics for the single and ensemble PCM models trained on the COX dataset

Algorithm		$R^{2}_{CV}$	RMSE_CV_	$R^{2}_{0\ test}$	RMSE_test_
A					
GBM		0.59	0.77	0.60	0.76
RF		0.60	0.78	0.61	0.79
SVM		0.61	0.75	0.60	0.76
B					
Greedy ensemble		–	0.73	0.63	0.73
Linear stacking		0.63	0.73	0.63	0.73
EN stacking		0.63	0.72	0.62	0.72
SVM linear stacking		0.63	0.73	0.62	0.73
SVM radial stacking		0.63	0.73	0.63	0.73
RF stacking		0.61	0.76	0.58	0.77

Combining single models trained with different algorithms in model ensembles allows to increase model predictive ability. We obtained the highest $R^{2}_{0\ test}$ and RMSE_test_ values namely, 0.63 and 0.73 pIC_50_ unit respectively, with the greedy ensemble, and with the following model stacking techniques: (1) linear, and (2) SVM radial.

*EN* Elastic Net, *GBM* Gradient Boosting Machine, *RF* Random Forest, *RMSE* root mean square error in prediction, *SVM* Support Vector Machines.

These models were subsequently combined into model ensembles using (1) greedy optimisation (1,000 iterations), and (2) model stacking (Table 3). The function *Validation* served to calculate the values for the statistical metrics on the test set. The observed against the predicted values on the test set were reported with the function *CorrelationPlot* (Fig. 3b).

All model ensembles displayed higher predictive power on the test set than single PCM models, except for RF Stacking (Table 3). The lowest RMSE value on the test set, namely 0.72 was obtained with the Elastic Network (EN) Stacking model (Table 3), whereas the highest $R^{2}_{0}$ value, namely 0.63, was obtained with the greedy, the Linear Stacking and the SVM Radial Stacking ensembles. As in the previous case study, these data indicate that higher predictive power can be obtained by combining single PCM models in more predictive model ensembles, although this improvement might be sometimes marginal. This case study illustrates the versatility of *camb* to train and validate PCM models from amino acid sequences and compound structures in an integrated and seamless modelling pipeline.

## Availability and future directions

*camb* is coded in R, C++, Python and Java and is available open source at https://github.com/cambDI/camb. To install *camb* from R type: library(devtools); install_github(“cambDI/camb/camb”). We plan to include further functionality based on the C++ Indigo API, and to implement new error estimation methods for regression and classification models. Additionally, we plan to further integrate the python library RDkit with *camb*. The package is fully documented and includes the usage examples and details of the R functions implemented in *camb*.

## Conclusions

In silico predictive models have proved valuable for the optimisation of compound potency, selectivity and safety profiles. In this context, *camb* provides an open framework to (1) compound standardisation, (2) molecular and protein descriptor calculation, (3) pre-processing and feature selection, model training, visualisation and validation, and (4) bioactivity/property prediction for new molecules. All the above functionalities will speed up model generation, provide reproducibility and tests of robustness. *camb* functions have been designed to meet the needs of both expert and amateur users. Therefore, *camb* can serve as an education platform for undergraduate, graduate, and post-doctoral students, while providing versatile functionalities for predictive bioactivity/property modelling in more advanced settings.

## Additional files

Additional file 1: QSPR with camb.pdf—Tutorial demonstrating the utility of the camb [1] package by presenting a pipeline which generates various aqueous solubility models using2D molecular descriptors calculated by the PaDEL-Descriptor package as input features. These models are then ensembled to create asingle model with a greater predictive accuracy. The trained ensemble is then put to use in making predictions for new molecules.

Additional file 2: PCM with camb.pdf—Tutorial demonstrating a pipeline to generate a Proteochemometric (PCM) model for mammal cyclooxygenase (COX) inhibitors. Further details about this dataset are reserved for a future publication. Similarly, the interested reader is referred to ref [1] and [2] forfurther details about PCM.
