# Supplementary material for: Chemically Aware Model Builder (camb): an R package for property and bioactivity modelling of small molecules
Source: J Cheminform. 2015 Aug 28;7:45. doi: 10.1186/s13321-015-0086-2 (PMC4551546; doi:10.1186/s13321-015-0086-2)
Supplement: Additional file 1: — QSPR with camb.pdf—Tutorial demonstrating the utility of the camb [1] package by presenting a pipeline which generates various aqueous solubility models using2D molecular descriptors calculated by the PaDEL-Descriptor package as input features. These models are then ensembled to create asingle model with a greater predictive accuracy. The trained ensemble is then put to use in making predictions for new molecules. [file 13321_2015_86_MOESM1_ESM.pdf]

QSPR with 'camb'
Chemically **A**ware **M**odel **B**uilder

Daniel S. Murrell*[1,5], Isidro Cortes-Ciriano†[2,5], Gerard J. P. van Westen[3], Ian P. Stott[4], Andreas Bender[1], Therese E. Malliavin[2], and Robert C. Glen[1]

[1] *Unilever Centre for Molecular Science Informatics, Department of Chemistry, University of Cambridge, Cambridge, United Kingdom.*
[2] *Unite de Bioinformatique Structurale, Institut Pasteur and CNRS UMR 3825, Structural Biology and Chemistry Department, 25-28, rue Dr. Roux, 75 724 Paris, France.*
[3] *ChEMBL Group, European Molecular Biology Laboratory European Bioinformatics Institute, Wellcome Trust Genome Campus, CB10 1SD, Hinxton, Cambridge, UK.*
[4] *Unilever Research, Bebington, UK.*
[5] *Equal contributors*

July 13, 2014

In the following sections, we demonstrate the utility of the `camb` [1] package by presenting a pipeline which generates various aqueous solubility models using 2D molecular descriptors calculated by the PaDEL-Descriptor package as input features. These models are then ensembled to create a single model with a greater predictive accuracy. The trained ensemble is then put to use in making predictions for new molecules.

Firstly, the package is loaded and the working directory set:

*dsmurrell@gmail.com
†isidrolauscher@gmail.com

```r
library(camb)
# setwd(path_to_working_directory)
```

1 Compounds

1.1 Reading and Preprocessing

The compounds are read in and standardised. Internally, Indigo's C API [2], incorporated into the `camb` package, is use to perform this task. Molecules are represented with implicit hydrogens, dearomatized, and passed through the InChI format to ensure that tautomers are represented by the same SMILES.

The `StandardiseMolecules` function allows representation of the molecular structures in a similarly processed form. The arguments of this function allow control over the maximum number of (i) fluorines, (ii) chlorines, (iii) bromines, and (iv) iodines the molecules can contain in order to be retained for training. Inorgnaic molecules (those containing atoms not in {H, C, N, O, P, S, F, Cl, Br, I}) are removed if the argument `remove.inorganic` is set to `TRUE`. This is the function's default behaviour. The upper and lower limits for molecular mass can be set with the arguments `min.mass.limit` and `max.mass.limit`. The name of the file containing the chemical structures is provided by the argument `structures.file`.

```r
std.options <- StandardiseMolecules(structures.file="solubility_2007_ref2.sdf",
                                    standardised.file="standardised.sdf",
                                    removed.file="removed.sdf",
                                    properties.file = "properties.csv",
                                    remove.inorganic=TRUE,
                                    fluorine.limit=3,
                                    chlorine.limit=3,
                                    bromine.limit=3,
                                    iodine.limit=3,
                                    min.mass.limit=20,
                                    max.mass.limit=900)
```

```
saveRDS(std.options, "standardisation_options.rds")
```

Molecules that Indigo manages to parse and that pass the filters are written to the file indicated by the `standardised.file` argument once they have been through the standardisation procedure. Molecules that were discarded are written to the file indicated by the `removed.file` argument. The molecule name and molecular properties specified in the structure file are written to the file indicated in the argument `properties.file` which is in CSV format. A column `kept` is added which indicates which molecules were deleted (0) or kept (1).

2 Target Visualisation

`camb` provides a function to visualise the density of the target variable. Visualising the distribution of the target variable gives can give a measure of how accurate a trained model is. The narrower the distribution the lower the RMSE should for the model to exhibit predictive power.

```
properties <- read.table("properties.csv", header=TRUE, sep="\t")
properties <- properties[properties$Kept==1, ]
head(properties)
targets <- data.frame(Name = properties$NAME, target = properties$EXPT)
p <- DensityResponse(targets$target) + xlab("LogS Target Distribution")
p
```

2.1 PaDEL Descriptors

One and two-dimensional descriptors can be calculated with the function `GeneratePadelDescriptors` provided by the PaDEL-Descriptor [3] Java library built into the `camb` package:

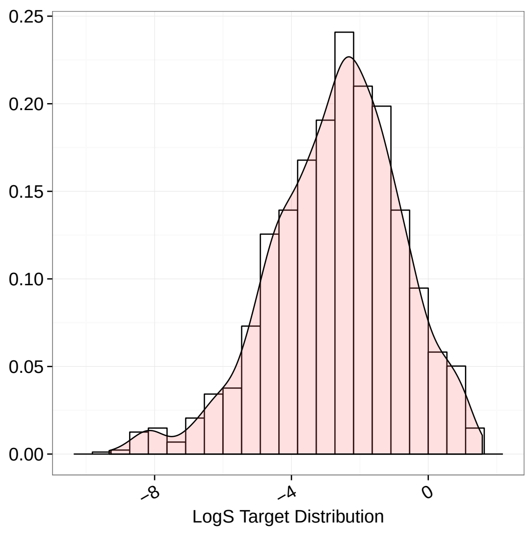

Figure 1: LogS Target Distribution

```
descriptor.types <- c("2D")
descriptors <- GeneratePadelDescriptors(standardised.file = "standardised.sdf",
    types = descriptor.types, threads = 1)
descriptors <- RemoveStandardisedPrefix(descriptors)
saveRDS(descriptors, file = "descriptors.rds")
```

3 Statistical Pre-processing

The descriptors and the target values are then merged by name into a single *data.frame*. We check that the number of rows of the merged and original *data.frames* are the same. We then split the *data.frame* into *ids*, x and y where *ids* are the molecule names, x is the block of descriptor values and y is the target values.

```
all <- merge(x = targets, y = descriptors, by = "Name")
ids <- all$Name
x <- all[3:ncol(all)]
y <- all$target
```

Sometimes, some descriptors are not calculated for all molecules, giving a "NA" or "Inf" as the descriptor value. Instead of removing that descriptor for all molecules, the missing descriptor values can be imputed from the corresponding descriptor values in the molecules that are closest to the molecule with the missing information. "Inf" descriptor values are first converted to "NA". For the imputation of missing descriptor values, the R package `impute` is required. Depending on the R version, it can be accessed from either `CRAN` or `Bioconductor`.

```
## Loading required package:  impute


## Cluster size 1606 broken into 375 1231
## Done cluster 375
## Done cluster 1231
```

The dataset is randomly split into a training set (80%) used for training and a holdout set (20%) which used to assess the predictive ability of the models on molecules drawn from the same distribution as the training set. Unhelpful descriptos are removed: (i) those with a variance close to zero (near-zero variance), and (ii) those highly correlated with one another:

```
dataset <- SplitSet(ids, x.imputed, y, percentage = 20)
dataset <- RemoveNearZeroVarianceFeatures(dataset,
    frequencyCutoff = 30)


## 397 features removed with variance below cutoff


dataset <- RemoveHighlyCorrelatedFeatures(dataset,
    correlationCutoff = 0.95)


## 121 features removed with correlation above cutoff
```

The descriptors are converted to z-scores by centering them to have a mean of zero and scaling them to have unit variance:

```
dataset <- PreProcess(dataset)
```

Five fold cross-validation (CV) is be used to optimize the hyperparameters of the models:

```
dataset <- GetCVTrainControl(dataset, folds = 5)
saveRDS(dataset, file = "dataset_logS_preprocessed.rds")
```

All models are trained with the same CV options, *i.e.* the arguments of the function `GetCVTrainControl`, to allow ensemble modeling as ensemble modelling requires the same data fold split over all methods used. It is important to mention that the functions presented in the previous code blocks depend on functions from the `caret` package, namely:

- RemoveNearZeroVarianceFeatures : nearZeroVar
- RemoveHighlyCorrelatedFeatures : findCorrelation
- PreProcess : preProcess
- GetCVTrainControl : trainControl

Experienced users might want to have more control over the underlying `caret` functions. This is certainly possible as the arguments given to the `camb` functions will be subsequently given to the `caret` counterparts in the typical ... R fashion. In fact, experienced users may want to learn from the internals of the functions `camb` provides and create their own specialised pipeline that fits their own modelling needs. `camb` is intended to speed up the modelling process and should not limit use of the extremely valuble caret package which `camb` utilises. The default values of these function permit the less experienced user to quickly handle the statistical preprocessing steps with ease, making a reasonable default choice for the argument values.

4 Model Training

In the following section we present the different steps required to train a QSPR model with `camb`. It should be noted that the above steps can be run locally on a low powered computer

such as a laptop and the preprocessed dataset saved to disk. This dataset can then be copied to a high powered machine or a farm with multiple cores for model training and the resulting models saved back to the local machine. Pro tip: Dropbox can be used to sync this proceedure so that manual transfer is not required.

```
dataset <- readRDS("dataset_logS_preprocessed.rds")
# register the number of cores to use in training
registerDoMC(cores = 10)
```

4.1 Support Vector Machines (SVM)

Firstly, a SVM using a radial basis function kernel is trained [4]. An base 2 exponential grid is used to optimize over the hyperparameters. The `train` function from the `caret` package is used directly for model training.

```
library(kernlab)
method <- "svmRadial"
tune.grid <- expand.grid(.sigma = expGrid(-8, 4, 2,
    2), .C = c(1e-04, 0.001, 0.01, 0.1, 1, 10, 100))
model <- train(dataset$x.train, dataset$y.train, method,
    tuneGrid = tune.grid, trControl = dataset$trControl)
saveRDS(model, file = paste(method, ".rds", sep = ""))
```

4.2 Random Forest

We proceed similarly in the case of a random forest (RF) model [5]. Here, only the mtry parameter needs optimisation.

```
library(randomForest)
method <- "rf"
tune.grid <- expand.grid(.mtry = seq(5, 100, 5))
```

```r
model <- train(dataset$x.train, dataset$y.train, method,
    tuneGrid = tune.grid, trControl = dataset$trControl)
saveRDS(model, file = paste(method, ".rds", sep = ""))
```

4.3 Gradient Boosting Machine

A gradient boosting machine (GBM) model [6] is trained optimising over the number of trees and the interaction depth.

```r
library(gbm)
method <- "gbm"
tune.grid <- expand.grid(.n.trees = c(500, 1000), .interaction.depth = c(25),
    .shrinkage = c(0.01, 0.02, 0.04, 0.08))
model <- train(dataset$x.train, dataset$y.train, method,
    tuneGrid = tune.grid, trControl = dataset$trControl)
saveRDS(model, file = paste(method, ".rds", sep = ""))
```

For each model we determine if our hyper-parameter search needs to be altered. In the following we focus on the RF model, though the same steps are also applied to the GBM and SVM models. If the hyper-parameters scanned lead you to what looks like a global minimum then you can stop scanning the space of hyper-parameters, otherwise you need to adjust the grid and retrain your model.

```r
model <- readRDS("rf.rds")
plot(model, metric = "RMSE")
```

5 Model Evaluation

Once the models are trained, the cross validated metrics for the optimised hyper-parameters become visible:

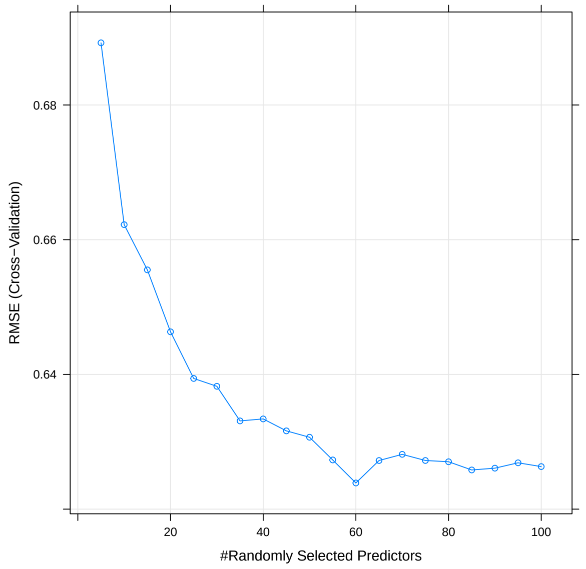

Figure 2: CV RMSE over the mtry hyperparameter for the RF

```
print(RMSE_CV(model, digits = 3))

## [1] 0.624

print(Rsquared_CV(model, digits = 3))

## [1] 0.8895
```

On the basis of the soundness of the obtained models, assessed through the value of the cross-validated metrics, we proceed to predict the values for the external (hold-out) set:

```
holdout.predictions <- as.vector(predict(model$finalModel,
    newdata = dataset$x.holdout))
```

To visualize the correlation between predicted and observed values, we use the `CorrelationPlot` function:

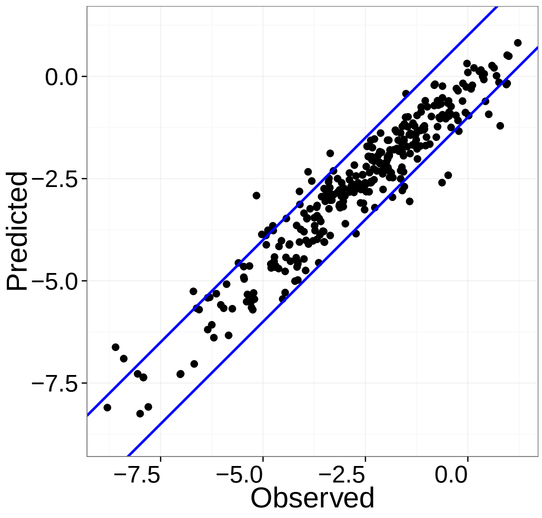

Figure 3: Observed vs Predicted

```
CorrelationPlot(pred = holdout.predictions, obs = dataset$y.holdout,
    PointSize = 3, ColMargin = "blue", TitleSize = 26,
    XAxisSize = 20, YAxisSize = 20, TitleAxesSize = 24,
    margin = 2, PointColor = "black", PointShape = 16,
    MarginWidth = 1, AngleLab = 0, xlab = "Observed",
    ylab = "Predicted")
```

6 Ensemble Modeling

In the following section, two ensemble modeling techniques are applied, namely greedy optimization and model stacking. Further information about these methods can be found in ref [7] and [8].

We append all the trained models to a list. The `sort` function shows the cross-validation RMSEs of the trained models in accending order.

```r
all.models <- list()
all.models[[length(all.models) + 1]] <- readRDS("gbm.rds")
all.models[[length(all.models) + 1]] <- readRDS("svmRadial.rds")
all.models[[length(all.models) + 1]] <- readRDS("rf.rds")

# sort the models from lowest to highest RMSE
names(all.models) <- sapply(all.models, function(x) x$method)
sort(sapply(all.models, function(x) min(as.vector(na.omit(x$results$RMSE)))))
```

```
##       gbm        rf svmRadial
##    0.5903    0.6239    0.6320
```

A greedy ensemble is then trained using 1000 iterations. The Greedy ensemble picks a linear combination of model outputs that is a local minimum in the RMSE landscape. The weights for each model can be seen in the `greedy$weights` variable. The RMSE of the greedy model can be found in the `greedy$error` variable.

```r
greedy <- caretEnsemble(all.models, iter = 1000)
```

```
## Loading required package:  pbapply
```

```r
sort(greedy$weights, decreasing = TRUE)
```

```
##       gbm svmRadial        rf
##     0.580     0.336     0.084
```

```r
saveRDS(greedy, file = "greedy.rds")
greedy$error
```

```
##   RMSE
## 0.5742
```

Similarly, we create a linear stack ensemble that uses the trained model inputs as input into the stack.

```
linear <- caretStack(all.models, method = "glm", trControl = trainControl(method = "cv"))
saveRDS(linear, file = "linear.rds")
linear$error
```

```
##   parameter  RMSE Rsquared  RMSESD RsquaredSD
## 1      none 0.574   0.9035 0.04047    0.01881
```

We also create a non-linear stack ensemble that uses the trained model inputs as input into the stack. In this case we use a Random Forest as the stacking model.

```
registerDoMC(cores = 1)
tune.grid <- expand.grid(.mtry = seq(1, length(all.models),
    1))
nonlinear <- caretStack(all.models, method = "rf",
    trControl = trainControl(method = "cv"), tune.grid = tune.grid)
```

```
## note: only 2 unique complexity parameters in default grid. Truncating the grid to 2 .
```

```
saveRDS(nonlinear, file = "nonlinear.rds")
nonlinear$error
```

```
##   mtry   RMSE Rsquared  RMSESD RsquaredSD
## 1    2 0.6107   0.8922 0.07509    0.02504
## 2    3 0.6195   0.8892 0.07569    0.02534
```

The greedy and the linear stack ensembles have a cross validated RMSEs that are lower than any of the individual models.

We then test to see if these ensemble models outperform the individual models on the holdout set.

```
preds <- data.frame(sapply(all.models, predict, newdata = dataset$x.holdout))
preds$ENS_greedy <- predict(greedy, newdata = dataset$x.holdout)
preds$ENS_linear <- predict(linear, newdata = dataset$x.holdout)
preds$ENS_nonlinear <- predict(nonlinear, newdata = dataset$x.holdout)
sort(sqrt(colMeans((preds - dataset$y.holdout)^2)))
```

```
##    ENS_linear    ENS_greedy           gbm ENS_nonlinear
##        0.5109        0.5126        0.5156        0.5515
##            rf     svmRadial
##        0.5938        0.5984
```

The linear ensemble slightly outperforms other models on the holdout set as well. This leads us to choose this as the most predictive model for future predictions. In the case that the ensemble models underperform the single models on the holdout set, it is advisable to pick the best single model for future predictions as a simpler model is a better model performance being equal.

7 External Predictions

One of the main attractions of this package is that it makes standardising and making predictions on new molecules a simple task. It is essential to ensure that the same standardisation options and descriptor types are used when the model is applied to make predictions for new molecules.

```
test_structures_file <- system.file("test_structures",
    "structures_10.sdf", package = "camb")
predictions <- PredictExternal(test_structures_file,
    std.options, descriptor.types, dataset, linear)
```

```
## [1] "Standardising Structures: Reading SDF (R)"
## [1] "Generating Descriptors"
```

```
print(predictions)
```

```
##         id prediction
## 1  B000088    -1.9469
## 2  B000139    -2.1535
## 3  B000236    -6.3559
## 4  B000310    -4.9248
## 5  B000728    -3.2997
## 6  B000785    -2.4975
## 7  B000821    -2.1867
## 8  B000826    -3.1167
## 9  B001153     0.4922
## 10 B001156    -1.1043
```

References

[1] D. S. Murrell, I. Cortes-Ciriano, G. J. van Westen, T. Malliavin, and A. Bender, "Chemistry Aware Model Builder (camb): an R package for predictive bioactivity modeling," *http://github.com/cambDI/camb*, 2014.

[2] Indigo, "Indigo cheminformatics library," 2013. [Online]. Available: `http://ggasoftware.com/opensource/indigo/`.

[3] C. W. Yap, "PaDEL-descriptor: an open source software to calculate molecular descriptors and fingerprints.," *J. Comput. Chem.*, vol. 32, pp. 1466–1474, 2011. [Online]. Available: `http://www.ncbi.nlm.nih.gov/pubmed/21425294`.

[4] A. Ben-Hur, C. S. Ong, S. Sonnenburg, B. Schlkopf, and G. Rtsch, "Support Vector Machines and Kernels for Computational Biology," *PLoS Computational Biology*, vol. 4, no. 10, F. Lewitter, Ed., e1000173, Oct. 2008.

[5] L. Breiman, "Random forests," *Machine Learning*, vol. 45, no. 1, pp. 5–32, Oct. 2001.

[6] J. H. Friedman, "Greedy function approximation: a gradient boosting machine.," *The Annals of Statistics*, vol. 29, no. 5, pp. 1189–1232, Mathematical Reviews number (MathSciNet) MR1873328, Zentralblatt MATH identifier01829052.

[7] Z. Mayer, "CaretEnsemble: framework for combining caret models into ensembles. [r package version 1.0]," 2013.

[8] R. Caruana, A. Niculescu-Mizil, G. Crew, and A. Ksikes, "Ensemble selection from libraries of models," in *Proceedings of the Twenty-first International Conference on Machine Learning*, ser. ICML '04, New York, NY, USA: ACM, 2004, p. 18.
