# Supplementary material for: Chemically Aware Model Builder (camb): an R package for property and bioactivity modelling of small molecules
Source: J Cheminform. 2015 Aug 28;7:45. doi: 10.1186/s13321-015-0086-2 (PMC4551546; doi:10.1186/s13321-015-0086-2)
Supplement: Additional file 2: — PCM with camb.pdf—Tutorial demonstrating a pipeline to generate a Proteochemometric (PCM) model for mammal cyclooxygenase (COX) inhibitors. Further details about this dataset are reserved for a future publication. Similarly, the interested reader is referred to ref [1] and [2] forfurther details about PCM. [file 13321_2015_86_MOESM2_ESM.pdf]

Proteochemometrics (PCM) with 'camb' **C**hemistry **A**ware **M**odel **B**uilder

Isidro Cortes-Ciriano*[1,5], Daniel S. Murrell†[2,5], Gerard J. P. van Westen[3], Ian P. Stott[4], Andreas Bender[2], Therese E. Malliavin[1], and Robert C. Glen[2]

[1] *Unite de Bioinformatique Structurale, Institut Pasteur and CNRS UMR 3825, Structural Biology and Chemistry Department, 25-28, rue Dr. Roux, 75 724 Paris, France.*

[2] *Unilever Centre for Molecular Science Informatics, Department of Chemistry, University of Cambridge, Cambridge, United Kingdom.*

[3] *ChEMBL Group, European Molecular Biology Laboratory European Bioinformatics Institute, Wellcome Trust Genome Campus, CB10 1SD, Hinxton, Cambridge, UK.*

[4] *Unilever Research, Bebington, UK.*

[5] *Equal contributors*

March 31, 2015

In the following sections, we present a pipeline to generate a Proteochemometric (PCM) model for mammal cyclooxigenase (COX) inhibitors. Further details about this dataset are reserved for a future publication. Similarly, the interested reader is referred to ref [1] and [2] for futher details about PCM.

Firstly, the package needs to be loaded and the working directory specified:

```
library(camb)
#setwd('path_to_working_directory')
```

*isidrolauscher@gmail.com

†dsmurrell@gmail.com

1 Compounds

1.1 Reading and Preprocessing

We proceed to read the compounds. Given that some smiles contain smarts patterns where the hash symbol is present, it is necessary to switch off the argument *comment.char* in order not to clip the smiles:

```
smiles <- read.table("smiles_COX.smi", header = FALSE,
    comment.char = c(""))
```

The function *StandardiseMolecules* enables the depiction of molecular structures in the same (standardised) form. The different arguments of this function allow control over the maximum number of (i) fluorines, (ii) chlorines, (iii) bromines, and (iv) iodines the molecules contains in order to be retained for training. Inorgnaic molecules (those containing atoms not in: {H, C, N, O, P, S, F, Cl, Br, I}) are removed if the argument "remove.inorganic" is set to "TRUE", which is the default value. Additionally, upper and lower limits for the molecular mass can be set with the arguments "min.mass.limit" and "max.mass.limit". The name of the file containing the chemical structures is input to the argument "structures.file".

```
StandardiseMolecules(structures.file="smiles_COX.smi",
standardised.file="standardised.sdf",
removed.file="removed.sdf",
remove.inorganic=TRUE,
fluorine.limit=-1,
chlorine.limit=-1,
bromine.limit=-1,
iodine.limit=-1,
min.mass.limit=-1, #suggested value 20
max.mass.limit=-1) #suggested value  900
```

The properties specified in the structure file of all molecules and an index, in the column "kept", indicating which molecules were deleted (0) and kept (1), are written to the file `"standardisation_info.csv"`. Molecules that Indigo manages to parse and that pass the filters are written to the file indicated in the argument "standardised.file". By contrast,

molecules that are discarded for training purposes are written to the file indicated in the argument "removed.file".

```
standardised_info <- read.table("standardisation_COX_info.csv",header=TRUE,sep="\t")
```

Default values of the arguments of the function "StandardiseMolecules" are not stringent. In the present case, all molecules are kept, thus all values in the columns "kept" are equal to "1". In the case of a SDF file, the function assumes that there is information for all molecules for all fields (properties). The function *StandardiseMolecules* generates an additional file, namely 'properties.csv', containint the molecule index and the aforesaid column "kept", which indicated if molecules were correctly standardized or not.
The values corresponding to an individual property in a ".sdf" file can be accessed with the function "GetPropertySDF". Similarly, the function "GetPropertiesSDF" retrieves the information for all properties of a given ".sdf" file. A data.frame with all properties is returned. The number of molecules from which the information has to be retrieved can be indicated with the argument `"number_processed"`. The default value for this argument is `"-1"`, which indicates that the properties will be extracted for all molecules in the input file.

```
ShowPropertiesSDF("test.sdf") # a mock .sdf file
GetPropertySDF("test.sdf",property="Name",
               number_processed=10)

all_properties <- GetPropertiesSDF("test.sdf",number_processed=10)
```

1.2 PaDEL Descriptors

One and two-dimensional PaDEL[3] descriptors and fingerprints can be calculated with the function "GeneratePadelDescriptors":

```
descriptors_COX <- GeneratePadelDescriptors( +
  standardised.file="smiles_COX.smi",threads = 1)

descriptors <- RemoveStandardisedPrefix(descriptors)
saveRDS(descriptors, file="Padel_COX.rds")
```

```
descriptors <- readRDS("Padel_COX.rds")
```

Sometimes, some descriptors are not calculated for all molecules, thus giving a "NA" or "Inf" as descriptor value. Instead of removing that descriptor for all molecules, the missing descriptor values can be imputed from the corresponding descriptor values of the rest of molecules. Descriptor values equal to "Inf" are converted to "NA". For the imputation of missing descriptor values, the R package *impute* is required. Depending on the R version, it can be accessed from either *CRAN* or *Bioconductor*.

```
descriptors <- ReplaceInfinitesWithNA(descriptors)
descriptors <- ImputeFeatures(descriptors)
```

1.3 Circular Morgan Fingerprints

The calculation of circular Morgan fingerprints requires the python library RDkit, given that the function "MorganFPs" calls a python script for the calculation of this type of fingerprints. The python code is available in the "extdata" folder of the package or at:
https://github.com/isidroc/FingerprintCalculator.
For a detailed discussion about circular Morgan fingerprints, we refer the interested reader to ref. [4]. When using integrated development environments (IDE) such as RStudio, the environment variables might not be defined within the R session. However, they can be redefined with the R function "Sys.setenv". In any case, the function "MorganFPs" requires this information in the arguments "PythonPath", path to python in the system, and "RDkitPath", the path to the RDkit library. For instance, the information of the latter is contained in the environment variable `$RDBASE` in Mac OS.

```
Sys.setenv(RDBASE="/usr/local/share/RDKit")
Sys.setenv(PYTHONPATH="/usr/local/lib/python2.7/site-packages")

fps_COX_512 <- MorganFPs(bits=512,radii=c(0,1,2),mols='smiles_COX.smi',
                         output='COX',keep='hashed_counts',
                         RDkitPath='/usr/local/share/RDKit',
                         PythonPath='/usr/local/lib/python2.7/site-packages',
                         images = FALSE, unhashed = FALSE,
```

```
                              extMols = FALSE, unhashedExt = FALSE,
                              logFile = FALSE)

saveRDS(fps_COX_512,file="fps_COX_512.rds")
fps_COX_512 <- readRDS("fps_COX_512.rds")
```

The function ’MorganFPs” enables the calculation of the following types of fingerprints:

- Hashed fingerprints in **binary format** of a given number of bits (argument ’bits’) considering substructures with a maximum radius, defined with the argument ’radius’, for the molecules specified in the argument ’mols’. These fingerprints are dropped to the output file `COX_hashed_binary.csv`, where output corresponds to the value of the argument ’output’ (”COX” in the example presented above). In the calculation of hashed fingerprints, several substructures can be mapped to the same bit position. Therefore, it might be important for the sake of interpretability to know which substructures are mapped to which bit in the fingerprint. This information is given in the file `COX_features_per_bit_hashed_fp.csv`. The first column of the file corresponds to the bit index, whereas the remaining columns correspond to substructure ids. This file is created automatically every time the function is run.

- Hashed fingerprints in **counts format** of a given number of bits (argument ’bits’) considering substructures of radius comprised in the set defined with the argument ’radii’, for the molecules specified in the argument ’mols’. These fingerprints are dropped to the output file `COX_hashed_counts.csv`.

- Unhashed fingerprints in **binary format**. All substructures present in the input molecules (argument ’mols’) are considered. Each position in the unhashed fingerprint corresponds to a given substructure. Thus, the resulting fingeprints are keyed. The smiles for each substructure and the number of atoms thereof is given in the output file `COX_smiles_substructures.csv`. This file is created automatically every time the function is run. Unhashed fingerprints in **binary format** are dropped to the output file `COX_unhashed_binary.csv`.

- Unhashed fingerprints in **counts format**. All substructures present in the input molecules (argument ’mols’) will be considered. Each position in the unhashed fingerprint corresponds to a given substructure. Thus, the resulting fingeprints are keyed.

In contrast to binary format, where a given bit is set on if a substructure appears in a molecule irrespective of the number of times the substructure is present therein, the number of occurrences of each substructure is accounted. The IDs and smiles for each substructure is given in the output file `COX_substructure_smiles_EXT.csv`, whereas the fingerprints are dropped to the output file `COX_unhashed_counts.csv`. The file `'COX_substructure_dictionary_EXT.csv'` indicates in which molecules each substructure appears. The first column of this file contains the substructure IDs, whereas the second the index (starting at zero) of the molecules harboring the substructures.

- In those cases where a given predictive model has been built on unhashed fingeprints, the same fingerprints should be calcualted for new molecules for which predictions are to be made by that model. To this aim, the function "MorganFPs" enables the calculation of both hashed and unhashed fingerprints (in both binary and counts format) for the molecules present in a given file (hereinafter referred to as external file). The unhashed fingeprints will be calculated based on the pool of substructures present in the file indicated in the argument "mols". To enable the calculation of fingerprints for the external file, the arguments "extMols", name of the external file, need to be set. The hashed fingerprints will be dropped to the following files:
  (i) binary format: `COX_hashed_binary_EXT.csv`,
  and (ii) counts format: `COX_hashed_counts_EXT.csv`. If the user also wants the calculation of unhashed fingerprints for the molecules present in the external file, the argument "unhashedExt" needs to be set to TRUE. In this case the unhashed fingeprints will be dropped to the following files:
  (i) binary format: `COX_unhashed_binary_EXT.csv`,
  and (ii) counts format: `COX_unhashed_counts_EXT.csv`.

The indexes of the molecules that could not be handled during the calculation are dropped to the file `incorrect_molecules_smiles_COX.csv`. Similarly, the indexes of the molecules from the external file that could not be processed are given in the file `incorrect_molecules_`+*root of the external file name* + —.csv.—

In the following paragraphs we describe in detail the arguments of the function "MorganFPs":

- **bits:** Number of bits of the hashed fingerprints. The default value is 512.
- **radii:** Substructure radii to be considered. A radius of 2 is equivalent to `ECFP-4`, where 4 corresponds to the diameter. The default value is the set 0,1,2. More information on

ECFP fingerprints can be found here:
`http://www.chemaxon.com/jchem/doc/user/ECFP.html`.

- **mols:** File containing the input molecules.
- **output:** Label that will be appended to all ouput files (see below).
- **keep:** The fingeprints that will be kept after the calculation. Apart from calculating different types of fingerprints, the function returns a data.frame with the type of fingerprints specified with this argument. Possible types are: `hashed_binary`,`hashed_counts`, `unhashed_binary`, `unhashed_counts`, and if applicable, `hashed_binaryEXT`, `hashed_countsEXT`, `unhashed_binaryEXT`, and `unhashed_countsEXT`. The default value is `"hashed_binary"`.
- **images:** If TRUE, individual ".pdf" files containing (i) the image of each substructure in the context of a molecule presenting it, and (ii) each molecule correctly processed, are created. Be aware that the number of substructures can be large depending on the number and diversity of the molecules present in the input file. Thus, allow for sufficient memory in those cases. The default value is FALSE.
- **unhashed:** If TRUE, unhashed fingeprints, both in binary format and with counts, are calculated. The default value is FALSE.
- **RDkitPath:** The path to the folder containing the RDkit library in your computer. On mac, the environment variable `$RDBASE` contains this information. The default value is "/usr/local/share/RDKit".
- **PythonPath:** Path to python (`$PYTHONPATH`).
The default value is "/usr/local/lib/python2.7/site-packages".
- **extMols:** If not FALSE, external file containing the molecules for which unhashed fingeprints are to be calculated with respect to the pool of substructures in the molecules present in the file specified in "mols". The default value is FALSE.
- **unhashedExt:** If TRUE, unhashed fingerprints are calcualted for the molecules specified in "extMols". The default value is FALSE.
- **logFile:** If not FALSE, file to which the log messages will be dropped. The default value is FALSE.

2 Targets

In the following section the tools provided by *camb* to calculate amino acid and whole protein descriptors will be presented.

2.1 Reading and Preprocessing Amino Acid Descriptors

In the current example, the amino acids implicated in ligand binding were extracted from the binding site of the ovine cyclooxygenase 1 (PDB ID: 3KK6). The corresponding amino acids for the rest of mammal cyclooxygenases were defined by sequence alignment. The amino acids corresponding to the target part of each compound-target combination in the dataset are given in the file `"AAs_COX.csv"`. Thus, the number of rows of this file corresponds to the number of datapoints in the dataset. We proceed to read the amino acids from the ".csv" file:

```
amino_acid_IDs <- read.table("AAs_COX.csv", sep = ",",
    header = TRUE, colClasses = c("character"), row.names = 1)
amino_acid_IDs <- amino_acid_IDs[, 2:ncol(amino_acid_IDs)]
```

Subsequently, 5 `Z-scales` will be calculated, which will serve to describe the target space in the PCM models. The descriptors will be saved to a ".rds" file.

```
amino_acid_IDs_zscales <- AADescs(Data = amino_acid_IDs,
    type = "Z5")
saveRDS(amino_acid_IDs_zscales, file = "Z5_COX.rds")
amino_acid_IDs_zscales <- readRDS("Z5_COX.rds")
```

The function `"AADescs"` permits the calculation of the following types of amino acid descriptors (futher details about these amino acid descriptors can be found in refs [5] and [6]):

- ProtFP8
- T-Scales
- VHSE
- ST-Scales

- BLOSUM
- FASGAI
- MSWHIM
- 5 Z-Scales
- 3 Z-Scales

This function outputs a data.frame which columns are indexed by the descriptors, and rows by the datapoints. Thus, the number of rows in the original data.frame or matrix, and the number of rows of the ouput data.frame are equal. If several descriptor types are chosen, by providing a vector of strings to the argument "type", descriptors are concatenated for the ease of further modeling. For instance:

```
AADescs(Data = c("Ala"), type = c("Z5", "BLOSUM"))

##  BLOSUM_1_Ala1  BLOSUM_2_Ala1  BLOSUM_3_Ala1  BLOSUM_4_Ala1
##           0.08          -0.92           0.53           0.00
##  BLOSUM_5_Ala1  BLOSUM_6_Ala1  BLOSUM_7_Ala1  BLOSUM_8_Ala1
##           0.24           0.19           0.66          -0.05
##  BLOSUM_9_Ala1 BLOSUM_10_Ala1      Z5_1_Ala1      Z5_2_Ala1
##           1.36           0.33           0.24          -2.32
##      Z5_3_Ala1      Z5_4_Ala1      Z5_5_Ala1
##           0.60          -0.14           1.30
```

Column names indicate the amino acid position in the original input data, and the type of descriptor.
Whole sequence descriptors can be computed with the function "SeqDescs". The function takes as argument either a UniProt identifier, or either a matrix or dataframe with the protein sequences. If a UniProt identifier is provided, the function gets firstly the sequence and then calculates the descriptors on the sequence.

```
Seq_descriptors_P00374 <- SeqDescs("P00374", UniProtID = TRUE,
    type = c("AAC", "DC"))
```

The available types of whole sequence descriptors are:[7]

- Amino Acid Composition ("AAC")
- Dipeptide Composition ("DC")
- Tripeptide Composition ("TC")
- Normalized Moreau-Broto Autocorrelation ("MoreauBroto")
- Moran Autocorrelation ("Moran")
- Geary Autocorrelation ("Geary")
- CTD (Composition/Transition/Distribution) ("CTD")
- Conjoint Traid ("CTriad")
- Sequence Order Coupling Number ("SOCN")
- Quasi-sequence Order Descriptors ("QSO")
- Pseudo Amino Acid Composition ("PACC")
- Amphiphilic Pseudo Amino Acid Composition ("APAAC")

2.2 Reading Dataset Information

In the following subsection, the file containing the information about the dataset, namely: target names, bioctivities, etc.., wil be read. Note that when reading molecules in smiles format from a ".csv" file into an R dataframe, the smiles are clipped after a hash ("#") symbol. A good practice is thus to also keep the smiles alone in a separate {.smi,.smiles} file.

```
dataset <- readRDS("COX_dataset_info.rds")
bioactivity <- dataset$standard_value
```

The bioactivity is in nM. We convert it to pIC50. To do that, we multiply by `10^-9` to convert the bioactivity units to M. Subsequently, the negative logarithm to base 10 is calculated:

```
bioactivity <- bioactivity * 10^-9
bioactivity <- -log(bioactivity, base = 10)
```

3 Dataset Visualization

Compounds can be depicted with the function "PlotMolecules". This function returns a list of four plots. Additionally, plots can also be written into a ".pdf" file if the argument "pdf.file" is not NULL. The argument "IDs" corresponds to the index of the molecules in the input file which are to be depicted. The name of the molecule in the input file will be used as the title of the image if the argument "useNameAsTitle" is set to TRUE. When using integrated development environments (IDE) such as RStudio, there might be errors with the rendering of the plots. In these cases, we adivse to use R in the command line.

```
plot_molecules <- PlotMolecules(sdf.file = "standardised.sdf",
    IDs = c(1, 2, 3, 4), pdf.file = NULL, useNameAsTitle = TRUE,
    PDFMain = NULL)

## [1] 1
## [1] 2
## [1] 3
## [1] 4

print(plot_molecules[[1]])
```

The distribution of the response variable can be explored with the function "DensityResponse" in the following way:

```
dens_resp <- DensityResponse(bioactivity, xlab = "pIC50",
    main = "", ylab = "Densitiy", TitleSize = 30, XAxisSize = 22,
    YAxisSize = 22, TitleAxesSize = 24, AngleLab = 0,
    lmar = 0, rmar = 0, bmar = 0, tmar = 0, binwidth = 0.3)
```

Given that the output of the function "DensityResponse" is a ggplot2 object, additional layers can be added to further customize the image.

A common analysis in bio- and chemoinformatics is to run a Pricipal Component Analysis (PCA) on either compound or target descriptors. The function "PCA" enables the calculation of the Principal Components (PCs) for a given set of descriptors. The function takes as arguments the descriptors and, optionally, the names of the rows, *i.e.* datapoints. Further arguments of the function *prcomp* from the package *stats*, use to run the PCA analysis, can be additionally set. The function returns a list with following elements :

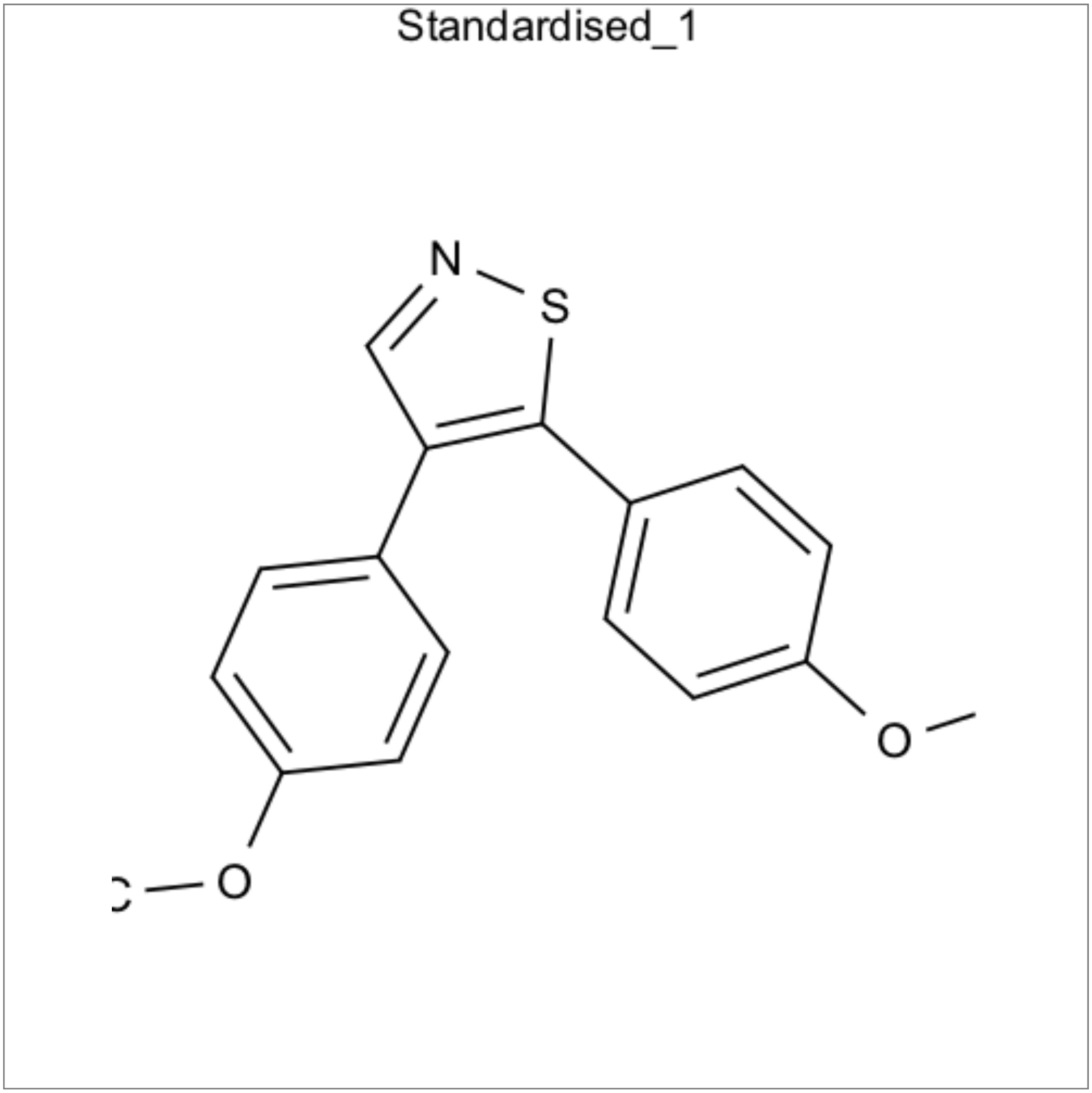

Figure 1: Example of compoud depiction.

- Data : a dataframe containing the two first PCs and the row names if provided. Rows are indexed as in the input data corresponds to a list.
- `PCs_All` : a dataframe containing all PCs.
- Std : a vector containing the standard deviation of all PCs.
- Info : information about the PCA analysis, such as the proportion of variance explained by each PC. It is always advisable to verify that the two or three first PCs explain a large proportion of the variance in the data, if conclusions are to be extracted from this type of analysis.

The function ”PCAPlot” provides an easy way to plot the first two PC. As all the plotting function provided with *camb*, it is based on *ggplot2*, which allows further customization by the user. Below is an example of how to use these two function to do a PCA analysis of the target space, which in this case is quantified by the amino acid descriptors of the amino acids present in the binding site of mammal cyclooxygenases.

```
target_PCA <- PCA(Data=amino_acid_IDs_zscales,
                  RowNames=dataset$accession,
                  cor=TRUE,scale = TRUE,
                  center = TRUE)

plot_PCA_COX <- PCAPlot(target_PCA$Data,PointSize=10,main="",
                  TitleSize=30,XAxisSize=20,YAxisSize=20,
                  TitleAxesSize=28,LegendPosition="bottom",
                  RowLegend=3,ColLegend=5,LegendTitleSize=15,
                  LegendTextSize=15)
```

Similarly, the chemical space can be explored by calculating pairwise compound similarities based upon compound descriptors. In this case, we use the Jaccard metric to calculate the distance between compounds. See the documentation of the *vegan* package for further information about other dissimilarity indices available. The function ”PairwiseDist” is based on the function ”vegdist” from the package *vegan*. Further arguments specified in the function ”PairwiseDist” will be passed to the function ”vegdist”. For illustration, the argument ”na.rm” will be given below to the ”PairwiseDist” function, which will be in turn passed to the function ”vegdist”.

```
pw_dist_comp_fps <- PairwiseDist(fps_COX_512, method = "jaccard",
    na.rm = TRUE)
saveRDS(pw_dist_comp_fps, file = "pairwise_dist_COX.rds")
```

The dissimilarity distribution can be depicted with the function "PairwiseDistPlot":

```
pw_dist_comp_fps <- readRDS("pairwise_dist_COX.rds")
plot_pwd <- PairwiseDistPlot(pw_dist_comp_fps, xlab = "Jaccard Index",
    ylab = "Density", TitleSize = 26, XAxisSize = 20,
    YAxisSize = 20, TitleAxesSize = 24, lmar = 0, rmar = 0,
    bmar = 0, tmar = 0, AngleLab = 0)
```

```
grid.arrange(dens_resp, plot_pwd, nrow = 2)
```

Before any modeling attempt, it is interesting to know which is the maximum performance achievable *on the basis* of the uncertainty of the available data.[8] To this aim, we consider the experimental uncertainty and the size of our dataset. In this case, a Gaussian Process (GP) model was trained in Matlab (data not shown) where the experimental uncertainty was optimized as a hyperparameter. The obtained value was 0.60 pIC50 units. This value is in accordance with the recently published value of 0.68 pIC50 units for public IC50 data.[9] With the function 'MaxPerf', we can calculate the maximum achievable performance, whereas with the function "MinPerf", we can calculate the minimum achievable performance.

```
max_performance <- MaxPerf(meanNoise=0,sdNoise=0.6,
                           resp=bioactivity, stds=NULL,
                           lenPred=length(bioactivity)*0.3,tmar = 0.2,
                           bmar = 0.2, rmar = 0.2,
                           lmar = 0.2)
grid.arrange(max_performance$p1,max_performance$p2,
             max_performance$p3,max_performance$p4,nrow=2)
```

In cases where the experimental uncertainty of the bioactivity values is not known, a sample from a Gaussian distribution, with mean equal to "meanNoise" and standard deviation equal to "sdNoise", is drawn. This sample will be considered as the experimental uncertainty of the bioactivity values. On the other hand, if the experimental uncertainties are known, they

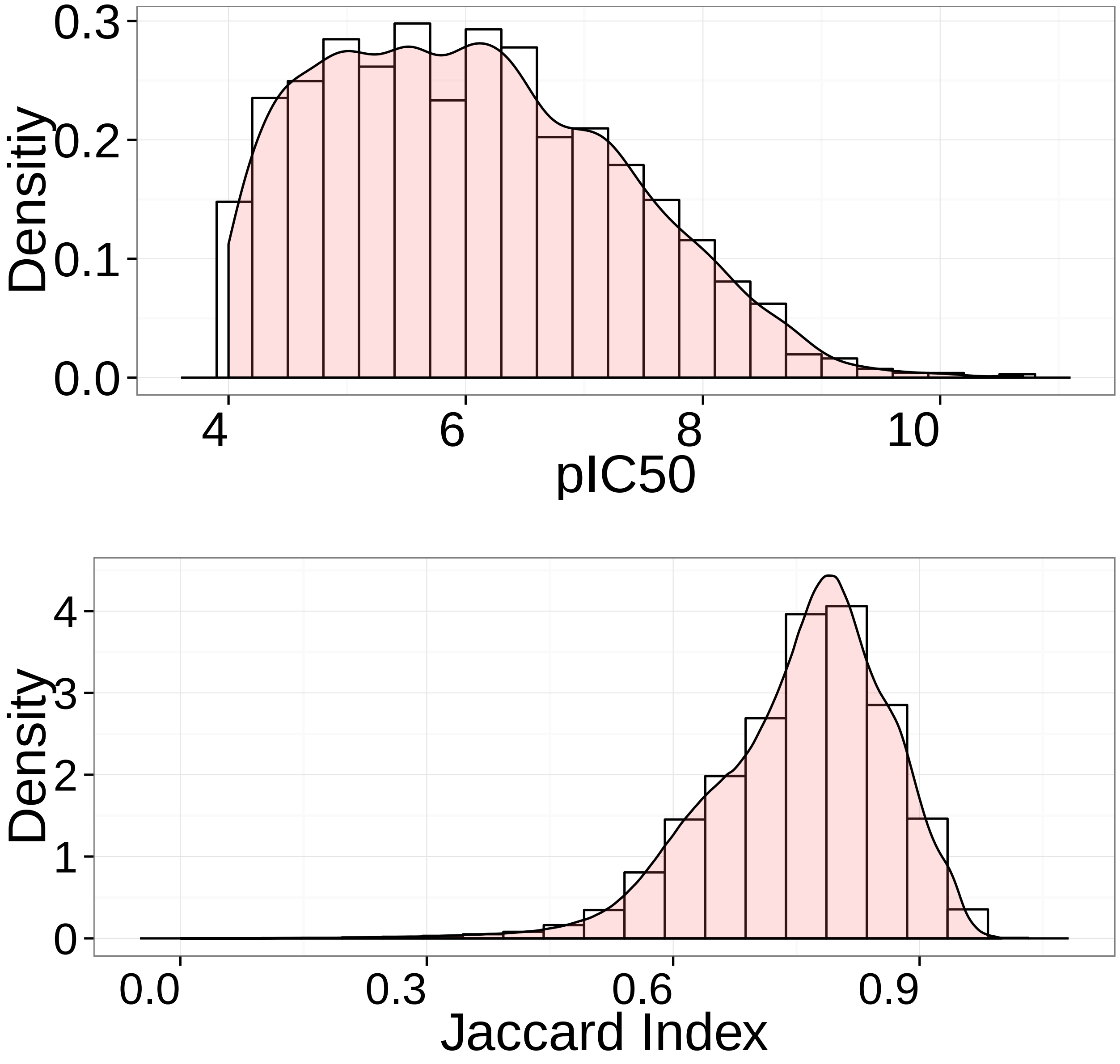

Figure 2: Density of the response variable (upper panel). Pairwise Compound Jaccard Similarity (bottom pannel)

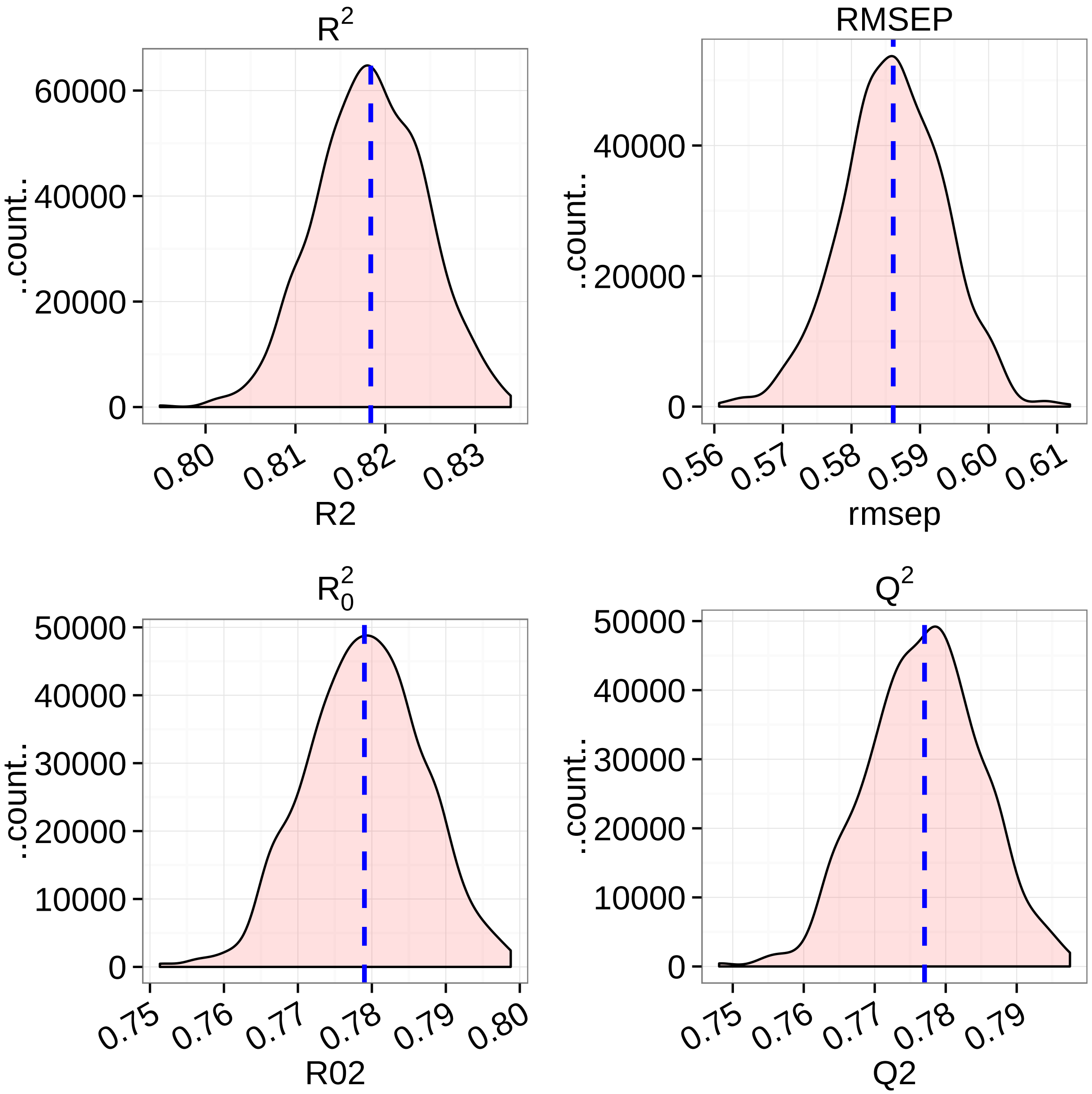

Figure 3: Maximum achievable model performance

can be input to the function via the argument "stds". The same applies to the function "MinPerf". The function returns a list of four plots, to which further layers can be added given that they are *ggplot* objects. Some graphical arguments are already available in the function "MaxPerf". In the example given above, the margin of the plots is controlled with the arguments "tmar", "bmar", "rmar", and "lmar", which respectively correspond to the top, bottom, right and left margins. As we can see from Figure 3, the maxima achievable values for the correlation metrics are far from one. Hence the importance of assessing the theoretical maximum performance the models can achieve on the basis of the uncertainty of the experimental measurements.

4 Statistical Pre-processing

Bioactivity annotations in ChEMBL are sometimes redundant, meaning that for a given target-compound combination there are more than one bioactivity values. To avoid this issue, we will remove redundant pairs and will keep the mean bioactivity value for those repeated compound-target combinations. Further information in this respect will appear in a future publication. In order to remove duplicate values, we run the file `"remove_duplicates.R"`:

```
source("remove_duplicates.R")
```

The dataset without repeated repetitions will be saved to the file `"Whole_dataset.rds"`. The dataset with only (i) compound descriptors, PaDEL and Morgan fingerprints, and (ii) amino acid descriptors will be saved to the files `"Whole_dataset_compound_descriptors.rds"` and `"Whole_dataset_aa_descriptors.rds"` respectively. All variables created while running the file `"remove_duplicates.R"` will be saved to the file `"data_processing_repetitions_COX.RData"`.

In cases where no repeated bioactivities are present for the same datapoint, the different descriptors blocks (PaDEL, Morgan fingerprints and amino acid descriptors in this case) can be simply stacked horizontally with the function "cbind".

Subsequently, we load the dataset without repetitions generated in the previous step. In addition, we remove those columns not containing descriptors (e.g. compound names):

```r
dataset <- readRDS("Whole_dataset.rds")
killset <- expression(c(tid, pref_name, accession,
    organism, chembl_id, standard_value, standard_units,
    standard_type, chembl_id.1, Name, Name.1, Name.2,
    rows))
bioactivity <- dataset$standard_value
compound_IDs <- dataset$chembl_id.1
dataset <- subset(dataset, select = -eval(killset))
```

Subsequently, we split the dataset into training (70%) and hold-out (external; 30%) sets that will be used to assess the predictive ability of the models. Furthermore, we remove the following descriptors: (i) those with a variance close to zero (near-zero variance), and (ii) those highly correlated:

```r
# Split the dataset into a training and holdout set
dataset <- SplitSet(compound_IDs, dataset, bioactivity,
    percentage = 30, seed = 1)

# Remove the descriptors that are highly correlated
# or have low variance
dataset <- RemoveNearZeroVarianceFeatures(dataset,
    frequencyCutoff = 30/1)
dataset <- RemoveHighlyCorrelatedFeatures(dataset)
```

We convert the descriptors to z-scores by centering them to zero mean and scaling their values to unit variance:

```r
dataset <- PreProcess(dataset)
```

Given that cross-validation (CV) will be used to optimize the hyperparameters of the models, we divide the training set in 5 folds:

```r
dataset <- GetCVTrainControl(dataset, seed = 1, folds = 5,
    repeats = 1, returnResamp = "none", returnData = FALSE,
    savePredictions = TRUE, verboseIter = TRUE, allowParallel = TRUE,
```

```
    index = createMultiFolds(y.train, k = folds, times = repeats))
saveRDS(dataset, file = "dataset_COX_preprocessed.rda")
```

All models are trained with the same CV options, *i.e.* the arguments of the function 'GetCV-TrainControl' to allow ensemble modeling (see below). It is important to mention that the functions presented in the previous code blocks depend on functions from the *caret* package, namely:

- RemoveNearZeroVarianceFeatures : nearZeroVar
- RemoveHighlyCorrelatedFeatures : findCorrelation
- PreProcess : preProcess
- GetCVTrainControl : trainControl

Experienced users might want to control more arguments of the underlying *caret* functions. This is certainly possible as the arguments given to the *camb* functions will be subsequently given to their *caret* counterparts. The default values of these function however permit the less experienced user to go through the statistical preprocessing steps with ease, though guaranteeing that the choice of the argument values is reasonable.

5 Model Training

In the following section we will present the different steps required to train a PCM model with *camb*.

```
dataset <- readRDS("dataset_COX_preprocessed.rda")
# Number of cores to be used during model training
cores <- 3
registerDoMC(cores)
```

5.1 Support Vector Machines (SVM)

Firstly, a SVM will be trained [10]. We define an exponential grid (base 2) to optimize the hyperparameters:

```
method <- "svmRadial"
exp_grid <- expGrid(power.from = -8, power.to = -6,
    power.by = 2, base = 2)
tune.grid <- expand.grid(.sigma = exp_grid)
```

Training (based on the *caret* function "train"):

```
modelCoxSVMrad <- train(dataset$x.train, dataset$y.train,
    method, tuneGrid = tune.grid, trControl = dataset$trControl)
saveRDS(modelCoxSVMrad, file = "model_SVM.rds")
```

5.2 Random Forest

We proceed similarly in the case of a random forest (RF) model[11].

```
method <- "rf"
modelCoxRF <- train(dataset$x.train, dataset$y.train,
    method, trControl = dataset$trControl)
saveRDS(modelCoxRF, file = "model_RF.rds")
```

5.3 Gradient Boosting Machine

We proceed similarly in the case of a gradient boosting machine (GBM) model[12].

```
method <- "gbm"
tune.grid <- expand.grid(.shrinkage = c(0.04, 0.08,
    0.12, 0.16), .n.trees = c(500), .interaction.depth = c(25))
modelCoxGBM <- train(dataset$x.train, dataset$y.train,
    method, tuneGrid = tune.grid, trControl = dataset$trControl)
saveRDS(modelCoxGBM, file = "model_GBM.rds")
```

6 Model Evaluation

Once the models are trained, the cross validated metrics can be calculated: we assume that the metric used for the choice of the best combination of hyperparameters is 'RMSE', which is normally considered as the aim of bioactivity modeling, *i.e.* how far (on average) are our predictions from the real bioactivity values?. In the following we focus on the RF model, though the same steps can be applied to the GBM and SVM models.

```
RMSE_CV_rf = RMSE_CV(modelCoxRF, digits = 3)
Rsquared_CV_rf = Rsquared_CV(modelCoxRF, digits = 3)
print(RMSE_CV_rf)

## [1] 0.775

print(Rsquared_CV_rf)

## [1] 0.5914135
```

On the basis of the soundness of the obtained models, assessed through the value of the cross-validated metrics, we proceed to predict the values for the external (hold-out) set:

```
holdout.predictions <- as.vector(predict(modelCoxRF$finalModel,
    newdata = dataset$x.holdout))
```

The predictive ability of the models is evaluated by the calculation of the following statistical metrics:
Internal validation:

$$q^2_{int} \ or \ R^2_{int} = 1 - \frac{\sum_{i=1}^{N_{tr}}(y_i - \widetilde{y}_i)^2}{\sum_{i=1}^{N_{tr}}(y_i - \bar{y}_{tr})^2} \tag{1}$$

$$RMSE_{int} = \frac{\sqrt{(y_i - \widetilde{y}_i)^2}}{N} \tag{2}$$

where N_{tr}, y_i, $\widetilde{y}_i$ and $\bar{y}_{tr}$ represent the size of the training set, the observed, the predicted and the averaged values of the dependent varia ble for those datapoints included in the training set. The ith position within the training set is defined by i.

External validation:

$$Q^2_{1\ test} = 1 - \frac{\sum_{j=1}^{N_{test}} (y_j - \widetilde{y}_j)^2}{\sum_{j=1}^{N_{test}} (y_j - \bar{y}_{tr})^2} \tag{3}$$

$$Q^2_{2\ test} = 1 - \frac{\sum_{j=1}^{N_{test}} (y_j - \widetilde{y}_j)^2}{\sum_{j=1}^{N_{test}} (y_j - \bar{y}_{test})^2} \tag{4}$$

$$Q^2_{3\ test} = 1 - \frac{[\sum_{j=1}^{N_{test}} (y_j - \widetilde{y}_j)^2]/N_{test}}{[\sum_{j=1}^{N_{tr}} (y_j - \bar{y}_{tr})^2]/N_{tr}} \tag{5}$$

$$RMSE_{test} = \frac{\sqrt{(y_j - \widetilde{y}_j)^2}}{N} \tag{6}$$

$$R_{test} = \frac{\sum_{j=1}^{N_{test}} (y_j - \bar{y}_{test})(\widetilde{y}_j - \bar{\widetilde{y}}_{test})}{\sqrt{\sum_{j=1}^{N_{test}} (y_j - \bar{y}_{test})^2 \sum (\widetilde{y}_j - \bar{\widetilde{y}}_{test})^2}} \tag{7}$$

$$R^2_{0\,test} = 1 - \frac{\sum_{j=1}^{N_{test}} (y_j - \widetilde{y}_j^{r0})^2}{\sum_{j=1}^{N_{test}} (y_j - \bar{y}_{test})^2} \tag{8}$$

where N_{tr}, N_{test}, y_j, $\widetilde{y}_j$, and $\bar{y}_{test}$ represent the size of the training and test sets, the observed, the predicted, and the averaged val ues of the dependent variable for those datapoints comprising the test set, respectively. $\bar{y}_{tr}$ represents the averaged values of the dependent variable for those datapoints comprising the training set. The *j*th position within the training set is defined by *j*.

$R^2_{0\,ext}$ is the square of the coefficient of determination through the origin, being $\widetilde{y}_j^{r0} = k\widetilde{y}_j$ the regression through the origin (observed versus predicted) and k its slope.
For a detailed discussion of both the evaluation of the predictive ability through the external set and about the three different formulations for Q^2_{ext}, namely $Q^2_{1\ ext}$, $Q^2_{2\ ext}$, and $Q^2_{3\ ext}$, see ref.[13]. To be considered as predictive, a model must satisfy the following criteria:[14, 15]

1. $q^2_{int} > 0.5$
2. $R^2_{ext} > 0.6$
3. $\frac{(R^2_{ext} - R^2_{0\,ext})}{R^2_{ext}} < 0.1$

4. $0.85 \leq k \leq 1.15$

The metrics for the external validation are given by:

```
MetricsRf <- Validation(predholdout.predictions, dataset$y.holdout,
    resp_tr = bioactivities)
```

The argument `"resp_tr"`, requires the bioactivity values of the datapoints present in the training set. These values are required by the metrics Q_1 and Q_3 (see above). Individual functions for all metrics presented above are also available in *camb*.

To visualize the correlation between predicted and observed values, we can use the 'CorrelationPlot' function:

```
CorrelationPlot(pred = holdout.predictions, obs = dataset$y.holdout,
    PointSize = 3, ColMargin = "blue", TitleSize = 26,
    XAxisSize = 20, YAxisSize = 20, TitleAxesSize = 24,
    margin = 2, PointColor = "black", PointShape = 16,
    MarginWidth = 1, AngleLab = 0, xlab = "Observed",
    ylab = "Predicted")
```

7 Ensemble Modeling

In the following section, two ensemble modeling techniques will be applied, namely greedy optimization and model stacking. Further information can be found in ref [16] and [17].

To get (i) the training set, (ii) the external set, (iii) the transformation applied to center and scale the descriptors before model training, and (iv) the model training options, we run the following lines of code:

```
data <- list()
attach(dataset)
data$transformation <- transformation
data$x.train <- x.train
data$y.train <- y.train
data$x.test <- x.holdout  # external set
data$y.test <- y.holdout  # external set
```

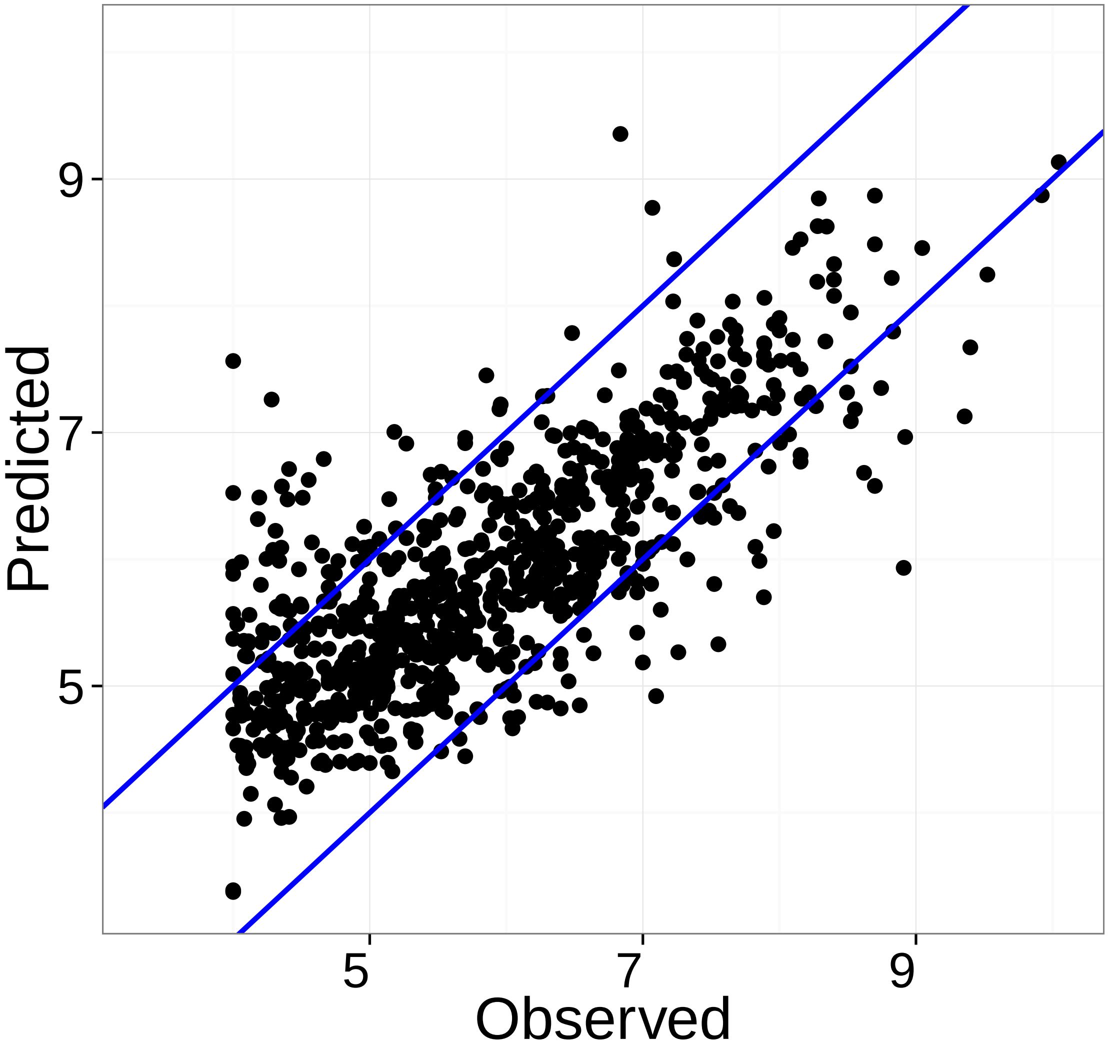

Figure 4: Observed vs Predicted

```
data$trControl <- trControl
saveRDS(data, file = "data_ensemble.rds")
detach(dataset)
```

Subsequently, we load the models previously trained. The list of models is in the file *modelsEnsemble*. Once all models have been loaded, we create the following ensemble:

```
greedy <- caretEnsemble(all.models, iter = 1000L)
sort(greedy$weights, decreasing = TRUE)

all.models <- list()
models <- as.vector(read.table("modelsEnsemble")$V1)

for (i in 1:length(models)) {
    model_load = paste("readRDS('", models[i], "')",
        sep = "")
    assign(paste("model_", i, sep = ""), eval(parse(text = model_load)))
    all.models[[length(all.models) + 1]] <- eval(parse(text = paste("model_",
        i, sep = "")))
}

names(all.models) <- sapply(all.models, function(x) x$method)
sort(sapply(all.models, function(x) min(as.vector(na.omit(x$results$RMSE)))))
```

Once all models have been loaded, we create the following ensemble:

```
greedy <- caretEnsemble(all.models, iter = 1000L)
sort(greedy$weights, decreasing = TRUE)
```

```
  # make a linear regression ensemble
  linear <- caretStack(all.models, method='glm')
summary(linear$ens_model$finalModel)

# make Elastic Net ensemble
```

```
enet_ens <- caretStack(all.models, method='enet')
coefs_enet_ens <- enet_ens$ens_model$finalModel$beta.pure +
[ncol(enet_ens$ens_model$finalModel$beta.pure)+1,]

# make SVM linear ensemble
trControl <- trainControl(method = "cv",  number=5)
tune.grid <- expand.grid(.C=expGrid(power.from=-14,
                                    power.to=10,power.by=1,base=2))
linear_svm <- caretStack(all.models, method='svmLinear',
                         trControl=trControl,tuneGrid=tune.grid)

# make SVM radial ensemble
trControl <- trainControl(method = "cv",  number=5)
tune.grid <- expand.grid(.sigma=expGrid(power.from=-14,
                                        power.to=10,power.by=1,base=2),
                         .C=expGrid(power.from=-14,power.to=10,
                                    power.by=2,base=2))
radial_svm <- caretStack(all.models, method='svmRadial',
                         trControl=trControl,tuneGrid=tune.grid)
```

We proceed to predict the bioactivities for the external (hold-out) set,

```
preds <- data.frame(sapply(all.models, predict, newdata=dataa$x.test))
preds$ENS_greedy <- predict(greedy, newdata=dataa$x.test)
preds$ENS_linear <- predict(linear, newdata=dataa$x.test)
preds$ENS_enet <- predict(enet_ens, newdata=x.test)
preds$ENS_SVMrad <- predict(radial_svm, newdata=x.test)c
preds$ENS_SVMlin <- predict(linear_svm, newdata=x.test)
```

and we calculate the values of the statistical metrics:

```
# Calculate metrics (We could also have applied
# Validation instead.)
Q2_1s <- apply(preds, 2, function(x) Qsquared1(x, dataa$y.test))
Q2_2s <- apply(preds, 2, function(x) Qsquared2(x, dataa$y.test))
```

```
Q2_3s <- apply(preds, 2, function(x) Qsquared3(x, dataa$y.test))
R2s <- apply(preds, 2, function(x) Rsquared(x, dataa$y.test))
R20s <- apply(preds, 2, function(x) Rsquared0(x, dataa$y.test))
RMSEs <- apply(preds, 2, function(x) RMSE(x, dataa$y.test))
```

References

[1] G. J. P. van Westen, J. K. Wegner, A. P. IJzerman, H. W. T. van Vlijmen, and A. Bender, "Proteochemometric modeling as a tool to design selective compounds and for extrapolating to novel targets," *Med. Chem. Commun.*, vol. 2, pp. 16–30, 1 2011.

[2] I. Cortes Ciriano, Q. U. Ain, V. Subramanian, E. B. Lenselink, O. Mendez Lucio, A. P. IJzerman, G. Wohlfahrt, P. Prusis, T. Malliavin, G. J. van Westen, and A. Bender, "Polypharmacology modelling using proteochemometrics: recent developments and future prospects," *In revision at Med. Chem. Comm*,

[3] C. W. Yap, "PaDEL-descriptor: an open source software to calculate molecular descriptors and fingerprints.," *J. Comput. Chem.*, vol. 32, pp. 1466–1474, 2011. [Online]. Available: `http://www.ncbi.nlm.nih.gov/pubmed/21425294`.

[4] D. Rogers and M. Hahn, "Extended-connectivity fingerprints.," *J. Chem. Inf. Model.*, vol. 50, no. 5, pp. 742–754, 2010.

[5] G. van Westen, R. Swier, J. K. Wegner, A. P. IJzerman, H. W. van Vlijmen, and A. Bender, "Benchmarking of protein descriptor sets in proteochemometric modeling (part 1): comparative study of 13 amino acid descriptor sets," *J. Cheminf.*, vol. 5, no. 1, p. 41, 2013.

[6] G. J. van Westen, R. F. Swier, J. K. Wegner, A. P. IJzerman, H. W. van Vlijmen, A. Bender, I. Cortes-Ciriano, J. P. Overington, G. J. P. V. Westen, and H. W. T. V. Vlijmen, "Benchmarking of protein descriptor sets in proteochemometric modeling (part 2): modeling performance of 13 amino acid descriptor sets," *Journal of Cheminformatics*, vol. 5, no. 1, p. 41, 2013.

[7] N. Xiao and Q. Xu, *Protr: protein sequence descriptor calculation and similarity computation with r*, R package version 0.2-1, 2014.

[8] I. Cortes Ciriano, G. J. P. van Westen, E. B. Lenselink, D. S. Murrell, A. Bender, and T. Malliavin, "Proteochemometrics Modeling in a Bayesian Framework," *J. Cheminf. Accepted*,

[9] T. Kalliokoski, C. Kramer, A. Vulpetti, and P. Gedeck, "Comparability of mixed IC50 data - a statistical analysis.," *PLoS One*, vol. 8, no. 4, e61007, 2013.

[10] A. Ben-Hur, C. S. Ong, S. Sonnenburg, B. Schlkopf, and G. Rtsch, "Support Vector Machines and Kernels for Computational Biology," *PLoS Computational Biology*, vol. 4, no. 10, F. Lewitter, Ed., e1000173, Oct. 2008.

[11] L. Breiman, "Random forests," *Machine Learning*, vol. 45, no. 1, pp. 5–32, Oct. 2001.

[12] J. H. Friedman, "Greedy function approximation: a gradient boosting machine.," *The Annals of Statistics*, vol. 29, no. 5, pp. 1189–1232, Mathematical Reviews number (MathSciNet) MR1873328, Zentralblatt MATH identifier01829052.

[13] V. Consonni, D. Ballabio, and R. Todeschini, "Evaluation of model predictive ability by external validation techniques," *J. Chemometrics*, vol. 24, no. 3-4, pp. 194–201, 2010.

[14] A. Golbraikh and A. Tropsha, "Beware of q2!," *J. Mol. Graph. Model.*, vol. 20, no. 4, pp. 269–276, Jan. 2002.

[15] A. Tropsha, P. Gramatica, and V. K. Gombar, "The Importance of Being Earnest: Validation is the Absolute Essential for Successful Application and Interpretation of QSPR Models," *QSAR Comb. Sci.*, vol. 22, no. 1, pp. 69–77, 2003.

[16] Z. Mayer, "CaretEnsemble: framework for combining caret models into ensembles. [r package version 1.0]," 2013.

[17] R. Caruana, A. Niculescu-Mizil, G. Crew, and A. Ksikes, "Ensemble selection from libraries of models," in *Proceedings of the Twenty-first International Conference on Machine Learning*, ser. ICML '04, New York, NY, USA: ACM, 2004, p. 18.
